# From pixels to pores: 3D-(im)printed hierarchically porous polymer monoliths

**DOI:** 10.1016/j.isci.2025.114539

**Published:** 2025-12-24

**Authors:** Benedikt Keitel, Simon Schimana, Amelie Huber, Yuki Yoshida, Hirotaka Shioji, Yoshitomo Furushima, Emine Billur Sevinis Ozbulut, Tomohiro Ohkawa, Shigeru Yoshimoto, Takashi Kubo, Hiroyuki Hosomi, Tsuyoshi Kato, Fumiya Uehara, Hiroko Futamura, Kana Nakanishi, Asuka Noda, Takashi Yamamoto, Boris Mizaikoff, Mehmet Dinc

**Affiliations:** 1 Hahn-Schickard, Sedanstraße 14, 89077 Ulm, Germany; 2 Institute of Analytical and Bioanalytical Chemistry, Ulm University, Albert-Einstein-Allee 11, 89081 Ulm, Germany; 3 Toray Industries Europe GmbH, Toray Automotive Center Europe, Am Gfild 6, 85375 Neufahrn bei Freising, Germany; 4 Toray Research Center, Inc., Shiga Laboratory, Otsu 520-8567, Shiga, Japan

**Keywords:** Chemistry, Molecular imprinted technique, Materials science, Polymers

## Abstract

Bridging molecular recognition with scalable materials is a central challenge in polymer science. Here, we present the first comprehensive characterization of LCD-based 3D-printed molecularly imprinted polymers (3DMIPs) with digitally programmable macrogeometries and tunable hierarchical porosity, revealing their optimization potential. As a case study, 3DMIPs targeting the specific enrichment of cannabidiol (CBD) are demonstrated. A highly porous lattice yields a 10.3-fold CBD enrichment, an imprinting factor of 3.7, and a CBD uptake of 1.65 mg/g, outperforming a coarser analog. Imaging and porosimetry reveal the pore architecture, pore interconnectivity, and pore size distribution, which, together with the macrogeometry, critically influence mass transfer and binding efficiency in these functional 3D materials. The 3DMIPs exhibit excellent thermal stability, highlighting suitability for practical applications. This work addresses the trade-off between molecular recognition, scalability, and design freedom, positioning 3DMIPs as a promising candidate for various applications, such as purifying health-promoting substances from complex plant matrices.

## Highlights

• Hierarchically porous, 3D-(im)printed monoliths enable specific CBD enrichment
• 3D printing concept for MIPs successfully transferred to therapeutic CBD
• First comprehensive characterization of 3DMIPs as a basis for material optimization
• Scalable, reproducible, and sustainable fabrication process for 3DMIPs

## Graphical abstract

**Figure:**
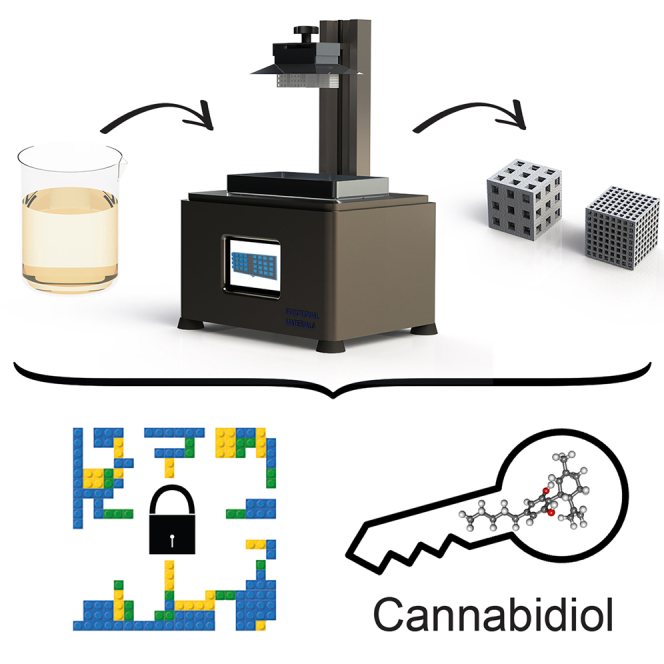

## Introduction

Molecularly imprinted polymers (MIPs) mimic natural recognition systems by forming organized, functional binding sites complementary to target substances.^1^ Ideally, MIP adsorbents combine antibody-like specificity and affinity with the inherent advantages of synthetic polymers, such as chemical and mechanical robustness and tunability.^2^^,^^3^^,^^4^ These characteristics render MIPs not only biomimetic but, in some aspects, even “biosuperior”, positioning them as promising candidates for applications ranging from separation and diagnostics to catalysis, drug delivery, and sensors.^5^^,^^6^^,^^7^ Despite their conceptual appeal and potential, however, widespread implementations and commercial scaling of MIPs are hindered, in particular by limitations in material and structural homogeneity and reproducibility.^8^^,^^9^^,^^10^^,^^11^

Straightforward conventional bulk polymerization methods for MIP fabrication are scalable but exhibit functional drawbacks: heterogeneous binding site distribution, template leaching, diffusion constraints (that is, slow mass transfer), and poor structural control (that is, irregular particle shapes and sizes after grinding).^12^^,^^13^ Consequently, research has pivoted toward more advanced imprinting techniques, such as direct polymerization techniques for micro- and nanoscale beads (e.g., suspension, emulsion, or precipitation polymerization) and surface imprinting.^8^^,^^9^^,^^14^^,^^15^ For example, hierarchically imprinted, methacrylate-based MIP beads with intrinsic mesoporosity and surface-confined binding sites have been reported to improve structural control and binding site accessibility.^16^^,^^17^^,^^18^ Halhalli et al. demonstrated surface-grafted, thin-walled imprinted polymer beads featuring accessible and uniform binding sites, which enhanced chromatographic performance regarding retentivity, enantioselectivity, and mass transfer.^19^^,^^20^ In this way, mass transfer properties and binding capacity can be optimized by fine-tuning the mesoporous or macroporous polymer structure and the surface-confined binding sites. While especially nanoscale MIPs address many performance-related issues through improved binding site accessibility and reduced heterogeneity, making them well suited for some analytical applications, their synthesis often involves complex multistep and low-yield procedures and suffers from batch-to-batch reproducibility challenges.^9^^,^^14^^,^^21^^,^^22^ On the macroscale, practical implementation is further complicated by high pressure drops in flow-through systems and difficulties in integrating nanomaterial formats (i.e., ease of handling).^23^^,^^24^^,^^25^

**Figure 1:**
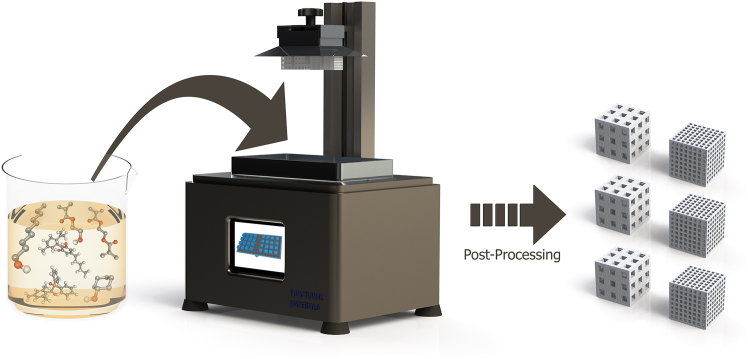
Cartoon-like representation of the photocurable resin, the scalable LCD-based 3D printing process, and the highly porous 3DMIPs with different macrogeometries

To overcome this trade-off between functionality, scalability, and applicability, we established a bottom-up liquid crystal display (LCD)-based 3D printing approach.^26^ This strategy integrates two self-assembly processes, noncovalent molecular imprinting and polymerization-induced phase separation (PIPS), into a single fabrication workflow. It enables the direct, emulsion-free fabrication of freestanding, hierarchically porous 3D-(im)printed polymer (3DMIP) monoliths with molecular recognition capabilities and digitally programmable macrogeometries (see Figure 1 for a cartoon-like visualization of the approach). By combining spatially resolved design with self-organized (sub-)micrometer porosity, the process offers unique control over structure-function relationships, enhancing mass transport while reducing pressure drop in monoliths — key parameters for future efficient operation in flow-through systems. Thus, integrating design freedom with scalable molecular recognition provides a novel route to tailor monolithic separation media properties in unprecedented ways. Notably, the process is compatible with low-cost, commercially available printers and partially contributes to sustainability according to the AGREEMIP sustainability assessment framework, as it generates no solid waste during fabrication (compared to traditional MIP syntheses).^26^^,^^27^ Hierarchical porosity arises from the interplay between defined macropores (i.e., pores resulting from spatially controlled photopolymerization guided by the pixels of the LCD-based printer) and phase-separation-driven (sub-)micrometer pores.^28^ Both pore types (the inherent and the printed porosity) are tunable via the print design or the resin formulation. Overall, this strategy offers an economical, scalable, and reproducible route to 3DMIP monoliths with application-ready geometries, and we hypothesize that the imprinting procedure can be transferred to other templates.

Building on our previous proof-of-principle study on 3DMIPs targeting an environmental pollutant, we now provide the first comprehensive analysis of the structure-function relationships in LCD-based 3DMIPs. As a case study for the transferability of the approach, we demonstrate 3DMIPs for the specific enrichment of the pharmaceutically relevant small molecule cannabidiol (CBD) for the first time. Despite its therapeutic potential, the industrial-scale purification of CBD from complex hemp raw extracts is inefficient.^29^ Conventional distillation methods often result in substantial CBD losses, whereas scalable alternatives require labor-intensive, multistep procedures or costly equipment.^29^ Hierarchically porous macroscopic 3DMIPs thus offer a promising route toward efficient and cost-effective CBD purification. However, such materials remain sparsely explored in the literature, particularly regarding systematic structure-function analysis. Here, we systematically analyze their adsorption performance, porosity, thermal stability, and mechanical properties, offering unprecedented insights into these advanced materials. These results provide a rationale for future material optimization and implementation in continuous purification processes.

## Results and discussion

To fabricate CBD (im)printed monoliths and their nonimprinted control polymers (NIPs), we formulated photopolymerizable, methacrylate-based resins engineered for hierarchical porosity and molecular recognition. In layer-by-layer 3D printing, the selection of monomer(s), initiator(s), and porogen(s) is key to achieving high molecular imprint fidelity, tailored morphology, and robust mechanical properties.^4^^,^^25^^,^^26^^,^^30^ The functional monomer 2-hydroxyethyl methacrylate (HEMA) and the crosslinker ethylene glycol dimethacrylate (EGDMA) were combined with the photoinitiator bis(2,4,6-trimethylbenzoyl)-phenylphosphine oxide (BAPO), selected for its spectral compatibility with the printer`s emission profile. Inherent porosity was introduced via a binary porogen system (3.6/1 v/v cyclohexanol/1-decanol), promoting phase separation during photopolymerization. The homogeneous resin was patterned into lattice cubes by digitally controlled photostructuring, yielding porous macroscopic polymers with a theoretical void volume of 50%, dictated by the porogen content in the resin.

Given the shared (meth)acrylate-based chemistry of commercial 3D printable resins and molecular building blocks commonly used in noncovalent molecular imprinting, we hypothesize that supplementing commercial resin formulations with tailored porogen systems provides a straightforward route toward 3DMIP synthesis.^31^^,^^32^^,^^33^

Following synthesis, we systematically evaluated the influence of computer-aided design (CAD)-defined macrogeometry, incubation time, and pH on CBD adsorption in batch binding studies. All results are benchmarked against NIPs synthesized with the same printing protocol and resin but in the absence of CBD to elucidate the templating effect and differences in adsorption performance.

### Binding behavior of the 3D polymer cubes for CBD

**Figure 2:**
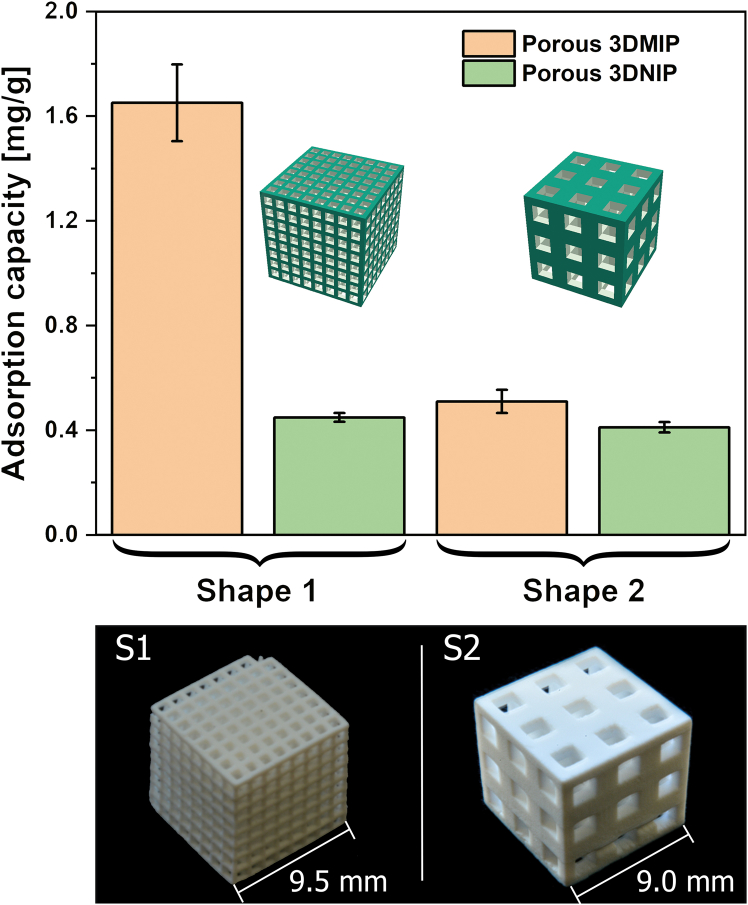
(Macro-)geometry-dependent binding behavior of porous 3DMIPs and 3DNIPs Two distinct lattice geometries — a delicate lattice (S1) and a bulkier structure (S2) — were evaluated for their adsorption capacity in batch binding experiments (60 min incubation with 0.0024 mmol CBD in 100 mL water/methanol 3/1 [v/v], followed by 15-min extraction in 3 mL ethanol). CBD content in the extraction solutions was quantified by UV/VIS spectroscopy. Data are represented as mean of three independent experiments (*n* = 3), and error bars represent the standard deviations. Photographs of the respective 3D-printed lattice structures are shown below the diagram.

Two distinct 3D lattice architectures were fabricated to assess how macroscopic design affects CBD adsorption: (1) a finely structured lattice matrix (S1) and (2) a coarse analog (S2). The S1-type 3DMIPs, characterized by narrow windows (760 μm) and thin polymer walls (380 μm), exhibited markedly superior performance in batch binding experiments with an imprinting factor (IF) of 3.7 and an adsorption capacity of 1.65 mg/g — more than 3-fold higher than its bulkier S2 counterpart (IF: 1.2; Q: 0.51 mg/g) (Figure 2). This superior binding behavior in S1-type 3DMIPs is attributed to shortened diffusion paths and increased accessibility of binding sites in the finer lattice architecture.

Furthermore, the S1 3DMIPs enabled an enrichment factor of 10.3, highlighting their applicability for efficient CBD extraction from aqueous-organic systems. Importantly, binding was reproducible, with a coefficient of variation lower than 9%. In contrast, the corresponding 3DNIPs showed negligible structure-dependent differences, supporting the presence of molecular imprinting effects and the absence of tailored binding sites (that is, nonspecific binding). These findings validate the design rationale for hierarchical 3D scaffolds to enhance mass transport and binding site accessibility in functional polymer systems.

Compared to the 3DMIPs presented here, nanoparticulate, methacrylate-based CBD imprinted polymers with adsorption capacities of up to 26.51 mg/g and an IF of 3.35 have been reported, indicating potential for future material optimization.^34^ Although the absolute capacities are lower than those of the best-performing nanoparticulate MIPs, the capacities of S1 and S2 3DMIPs still significantly surpass other reported CBD imprinted polymers with approximately 0.172 μmol/g (that is, 0.05 mg/g) for MIP particles and 148.05 ng/cm^3^ for MIP monoliths.^35^^,^^36^

**Figure 3:**
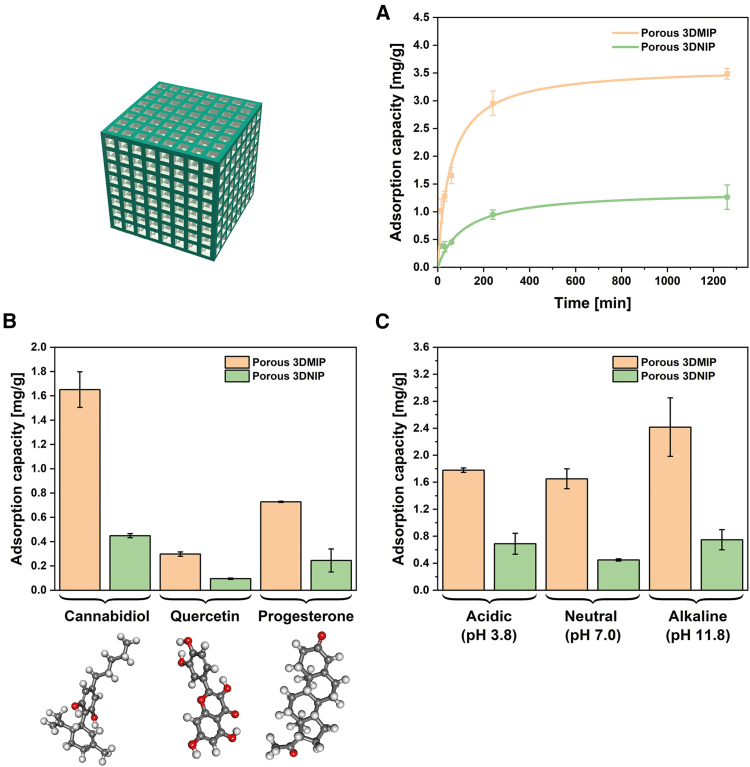
Characterization of CBD binding to porous S1-type 3DMIPs and their respective 3DNIPs in batch incubation studies The CBD content in the extraction solutions was quantified by UV/VIS spectroscopy. Data are represented as mean of three independent experiments (*n* = 3), and error bars represent the standard deviations. (A) CBD adsorption kinetics were investigated over 21 h (incubation: 0.0024 mmol CBD in 100 mL water/methanol 3/1 [v/v]; extraction in 3 mL ethanol for 15 min). The data were fitted using a pseudo-second-order kinetic model, yielding a rate constant k_2_ ≈ 0.0049 g/mg/min and equilibrium adsorption capacity Q_e_ ≈ 3.61 mg/g for 3DMIP and k_2_ ≈ 0.0092 g/mg/min and Q_e_ ≈ 1.31 mg/g for 3DNIP. See also Figure S1 for further information. (B) Specificity experiments performed under identical conditions with an incubation time of 60 min revealed preferential binding of CBD over quercetin and progesterone. (C) pH-dependent adsorption showed maximum CBD uptake under alkaline conditions (pH 11.8), with an adsorption capacity of 2.42 mg/g and an IF of 3.2.

Time-dependent adsorption studies (15 min to 21 h) confirmed the pronounced imprinting effect in the more efficient S1 3DMIPs, yielding IFs between 2.6 and 3.7. The MIPs exhibited a rapid initial CBD uptake, approaching equilibrium at approximately 3.5 mg/g after about 400 min, while the NIPs showed consistently lower adsorption capacities with a plateau of around 1.3 mg/g (see Figure 3A). These distinct kinetic profiles substantiate successful molecular imprinting and the presence of CBD binding sites in the 3DMIPs.

To gain deeper insight into these uptake mechanisms, we first modeled the adsorption kinetics. The pseudo-second-order model provided a superior fit compared to the pseudo-first-order model, consistent with previous reports on specific adsorption processes in noncovalent MIPs.^37^^,^^38^^,^^39^ For 3DMIPs, the pseudo-second-order model yielded a coefficient of determination (R^2^) of 0.976 and root-mean-square error (RMSE) of 0.151, compared to R^2^ = 0.933 and RMSE = 0.251 for the pseudo-first-order model. Similarly, for 3DNIPs, the pseudo-second-order model achieved R^2^ = 0.928 and RMSE = 0.096, while the pseudo-first-order model resulted in R^2^ = 0.875 and RMSE = 0.127. However, both models assume a fixed equilibrium adsorption capacity, which may not be valid in our finite volume system, where the CBD concentration decreases over time. Therefore, to complement these empirical kinetic fits, we additionally applied Crank’s diffusion model for adsorption from a stirred finite volume system into a plane sheet to derive the approximate apparent diffusion coefficients of both S1-type polymers (see supplemental information Figure S1).^40^ Using half the wall thickness of a cube pillar (190 μm) as the characteristic length scale yielded approximate apparent diffusion coefficients of D_3DMIP_ = 3.9 × 10^−13^ m^2^/s and D_3DNIP_ = 2.5 × 10^−13^ m^2^/s for the imprinted and nonimprinted polymers, respectively. These values confirm the enhanced mass transport properties of the 3DMIPs — approximately 1.6 times faster — and underscore the kinetic advantage achieved by molecular imprinting.

Additional binding experiments with S1-type polymers demonstrated a preferential binding of CBD over the flavonoid quercetin — naturally present in hemp and containing multiple hydroxyl groups — and over progesterone, a bioactive compound with the same molar mass and similar size, indicating specificity (see Figure 3B). Furthermore, pH-dependent experiments revealed consistently higher CBD uptake by 3DMIPs across all tested conditions, with a maximum of 2.42 mg/g (IF: 3.2) and an enrichment factor of 16.8 at pH 11.8 (Figure 3C). The enhanced adsorption at pH 11.8 results from the deprotonation of the phenolic hydroxyl groups of CBD, thereby enhancing their hydrogen bond acceptor capacity. Simultaneously, the aliphatic hydroxyl moieties of HEMA are expected to remain predominantly protonated at pH 11.8, acting as effective hydrogen bond donors and promoting interactions with deprotonated CBD. This pH sensitivity could be exploited in future real-world purification applications. Together, these findings confirm the transferability of the 3DMIP synthesis strategy from estradiol to other targets, exemplified by CBD.

### Porosity characterization of the macroscopic 3D polymers (S1 3DMIP vs. S1 3DNIP)

Having demonstrated the binding performance of the 3D functional polymers for CBD (that is, successful concept transfer), we next analyzed their structural characteristics to correlate morphology with performance. Key parameters included specific surface area, pore size distribution, and skeletal density.

Specific surface areas of crushed samples (3DMIPs and 3DNIPs, each as S1 and S2) were calculated from nitrogen sorption isotherms using the Brunauer-Emmett-Teller (BET) method (see supplemental information Figures S2, S3, and S4). Pore size distributions were subsequently derived from these sorption data by Grand Canonical Monte Carlo (GCMC) simulations (see supplemental information Figure S5).

The synthesized polymers exhibited specific surface areas ranging from approximately 80.5 to 95.2 m^2^/g (Table 1). Although the S1 3DNIP exhibited the highest surface area, its imprinted counterpart, S1 3DMIP, demonstrated superior adsorption capacity. This observation aligns with prior findings in porous 3DMIPs, emphasizing that adsorption efficiency depends not solely on surface area but also on diffusion pathways and binding site accessibility, both influenced by the macroscopic design.^26^ Indeed, even 3DMIPs with comparable specific surface areas but different lattice geometries showed markedly different binding behaviors, underscoring the critical importance of the macrostructural design in functional polymer systems.

**Table 1:** Specific surface areas and median pore sizes of the 3D polymers

Polymer type	Specific surface area (m^2^/g)	Median pore diameter (nm)
S1 3DMIP	87.6 ± 3.0	21 ± 1
S1 3DNIP	95.2 ± 3.2	18 ± 1
S2 3DMIP	91.6 ± 8.8	17 ± 1
S2 3DNIP	80.5 ± 7.7	17 ± 1

Determined by nitrogen sorption studies and calculated using the BET method. Data are represented as mean of two independent experiments (*n* = 2), and error bars represent the standard deviations. See also Figures S2, S3, and S4 for further information.

While a large surface area favors the provision of binding sites, the morphology of these functional materials significantly determines the diffusion and accessibility of the binding sites. Although nitrogen sorption provided insight into the mesoporous domain of the hierarchically structured materials (see Table 1; supplemental information Figure S5), its applicability is limited in the macroporous regime, especially for pores larger than 100 nm.^41^^,^^42^ To gain a deeper understanding of the morphological differences, we supplemented this analysis with mercury intrusion porosimetry and helium pycnometry on the better-performing S1-type polymers, thereby also taking macropores into account. It should be noted that although mercury intrusion porosimetry provides valuable information on pore size distribution and connectivity, high intrusion pressures can lead to local compaction, deformation, and pore collapse in mechanically soft or fragile polymer networks.

**Figure 4:**
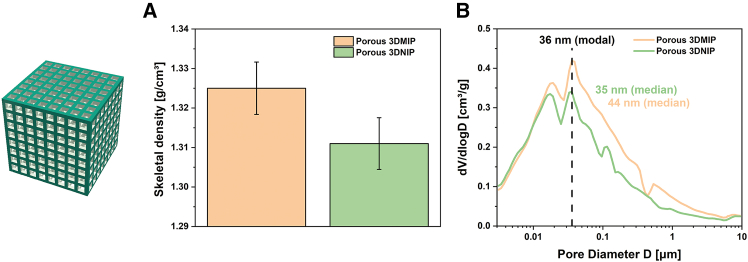
Microstructural differences between S1 3DMIP and S1 3DNIP (A) Skeletal densities determined by helium pycnometry. Lower skeletal density values suggest a higher fraction of inaccessible pores (i.e., blocked pores) in the NIP compared to the MIP. Error bars represent the instrumental error. (B) Pore size distributions obtained via mercury intrusion porosimetry reveal a greater abundance of small pores in the 3DNIP relative to the 3DMIP. Data are represented as mean of two independent experiments (*n* = 2).

Mercury intrusion porosimetry confirmed the presence of interconnected hierarchical porosity in these polymers, with calculated void volumes of 45% ± 1% for 3DMIP and 40% ± 1% for 3DNIP (according to Equation 6 in STAR Methods). Helium pycnometry suggested a trend toward marginally lower skeletal density in the 3DNIP, indicating the presence of a few inaccessible, isolated pores (see Figure 4A). Although the absolute difference in the skeletal densities is not interpreted as statistically significant, this trend aligns with the mercury intrusion porosimetry results, which revealed a less connected pore network in the 3DNIP. Therefore, the pycnometry data are interpreted as supporting evidence for isolated pores in the 3DNIP, contributing to its lower effective void volume (around 40% vs. theoretical 50%) and ultimately lower binding performance.

Pore size distributions from mercury intrusion porosimetry indicated a higher abundance of small pores in the NIPs, with a median pore diameter of 35 ± 2 nm and a specific pore volume of 0.507 ± 0.021 cm^3^/g, in contrast to 44 ± 2 nm and a specific pore volume of 0.609 ± 0.025 cm^3^/g for the MIPs (see Figure 4B). Both materials share a modal pore size of approximately 36 nm. These structural characteristics, including larger pore volume and greater pore accessibility in MIPs, are in agreement with a previous study on understanding nanoscale porosity in MIPs and affect mass transfer, leading to enhanced adsorption performance.^43^

However, the interpretation of porosimetry results must consider the “ink-bottle”. Narrow pore throats connecting larger internal voids can lead to an underestimation of internal cavity sizes. Also, a compaction or pore collapse at higher pressures would distort the measurement result. Accordingly, pore size data alone may not fully reflect the functional pore network, and complementary techniques remain essential for accurate pore structure analysis. Notably, the GCMC-derived trend in the mesopore distribution profile is similar to the mesopore regime obtained by mercury intrusion porosimetry, highlighting the complementary nature of these techniques.

**Figure 5:**
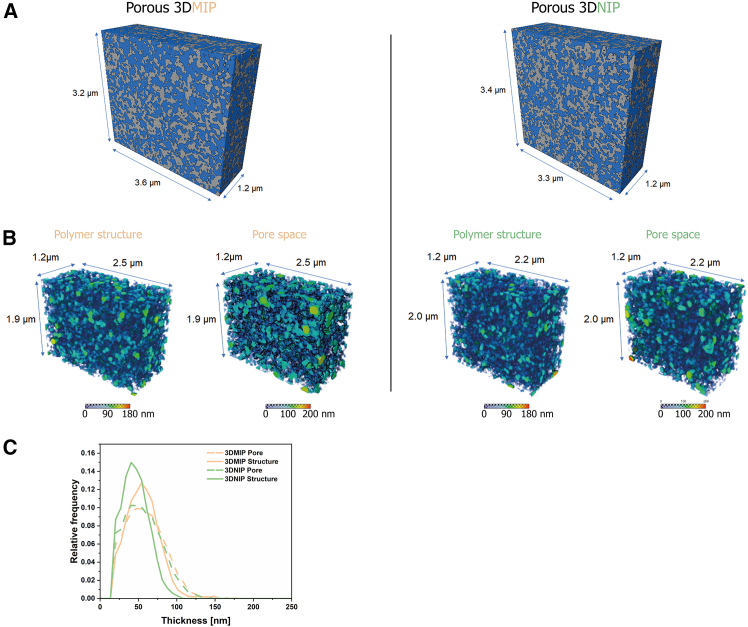
FIB/SEM tomography: 3D reconstruction and local thickness analysis (A) 3D reconstructions generated from FIB/SEM tomography data (gray: polymer matrix; blue: pores), depicting volumes of 13.82 μm^3^ for the 3DMIP (left) and 13.46 μm^3^ for the 3DNIP (right). They confirm the highly porous architecture and interconnected pore networks. See also videos S1 and S2 for further information. (B) 3D volumes illustrating the spatial distribution of pores and polymer in 3DMIP (left) and 3DNIP (right) with color-coded thicknesses as obtained from local thickness analysis. (C) Quantitative distributions of pore and polymer matrix thicknesses derived from local thickness analysis.

To validate the bulk porosity data and directly visualize the 3D pore architecture of the 3DMIP and 3DNIP, high-resolution focused ion beam/scanning electron microscopy (FIB/SEM) tomography was performed. “Slice-and-view” was conducted with a slice thickness of 3 nm within volumes of 3.6 × 3.2 × 1.2 μm^3^ (3DMIP) and 3.3 × 3.4 × 1.2 μm^3^ (3DNIP). Following alignment, binarization, and segmentation of the acquired image stacks, 3D reconstructions were generated (see Figure 5A as well as Videos S1 [3DMIP] and S2 [3DNIP]). The polymer matrix appears gray in the visualizations, while voxels representing the pore space are rendered in blue.

Video S1. FIB-SEM tomography of 3DMIP showing pore connectivity, related to Figure 5A

Video S2. FIB-SEM tomography of 3DNIP showing pore connectivity, related to Figure 5A

Quantitative analysis of the reconstructed volumes revealed porosities of 46% (3DMIP) and 51% (3DNIP). Despite exhibiting an inverse trend compared to mercury intrusion porosimetry, these values confirm the high inherent porosity of both polymers. Moreover, these values closely match the theoretical void volume of 50%, indicating high local pore interconnectivity. Indeed, approximately 99.8% of all detected pores in the analyzed volumes of 3DMIP and 3DNIP were part of a connected network, emphasizing the structural continuity of the porous architecture. The minor discrepancy between the results of mercury porosimetry and FIB/SEM tomography highlights the complementarity of bulk and local analyses in capturing the structural heterogeneity and complexity of the porous polymer networks.

Interestingly, Neusser et al. demonstrated that the interconnectivity of visualized pores in methacrylate-based MIPs prepared by bulk polymerization in acetonitrile/toluene is significantly higher compared to non-imprinted controls, along with increases of approximately 34% and 35% of the pore volume and pore surface area, respectively.^43^ In contrast, the 3DMIPs and 3DNIPs presented here exhibit similarly high interconnectivity and porosity values. This suggests that the printing process and the employed porogen system predominantly induce the porosity in the 3D-printed system.

To gain more detailed insight into the internal microstructure, we additionally conducted a quantitative local thickness analysis based on the segmented FIB/SEM data (see Figure 5B). This analysis revealed lower average wall thickness and pore size in the 3DNIP than in the 3DMIP, indicating a finer pore network in the former (see Figure 5C). Although the absolute pore sizes derived from thickness analysis are substantially smaller (maximum around 100 nm) than those determined by mercury intrusion porosimetry (which detects pores exceeding 1 μm), the relative trend remains consistent: 3DMIPs feature slightly larger pores than 3DNIPs. This discrepancy in absolute values arises from intrinsic methodological differences: while thickness analysis — based on the diameter of the largest inscribed sphere — underestimates the size of irregular or elongated pores, mercury intrusion quantifies the size of the narrowest pore throats based on the applied pressure. Collectively, these techniques provide unique insights into the hierarchically structured printed networks, elucidating the differences between 3DMIPs and 3DNIPs.

To complement the comprehensive material characterization and to investigate whether molecular preorganization in 3D-(im)printed hierarchically porous polymers leads to local variations in surface chemistry associated with the imprinted binding sites, time-of-flight secondary ion mass spectrometry (TOF-SIMS) was performed on a 3DMIP and 3DNIP. However, no significant differences in surface composition were observed between the two materials (see supplemental information Figures S6–S9, and S10), suggesting that the templated binding sites are distributed beyond the spatial resolution of TOF-SIMS. Future studies employing NanoSIMS, which offers higher spatial resolution, could provide a more detailed insight into the distribution of the recognition domains.

### Thermal evaluation and mechanical properties of the polymers

**Figure 6:**
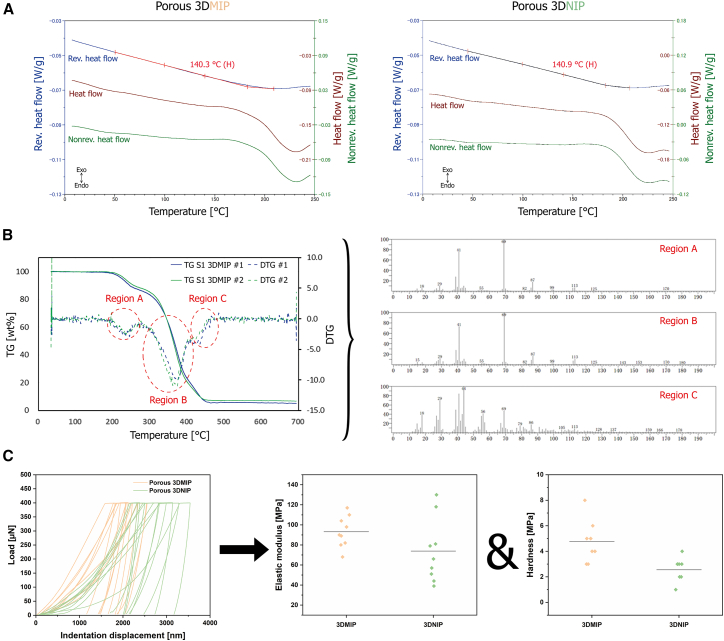
Thermal and mechanical characterization of 3DMIP and 3DNIP (A) TMDSC curves of 3DMIP (left) and 3DNIP (right) showing reversible heat flow, nonreversible heat flow, and heat flow (that is, the sum). Note: the reversible heat flow is plotted at three times the scale of the other signals. Both materials exhibit a broad glass transition region (reversible heat flow curve) with similar *T*_g_ values. *T*_g_ values are measured in two independent experiments (*n* = 2); a representative measurement is shown. (B) TG-MS analysis of 3DMIP reveals high thermal stability up to approximately 180 °C, followed by multistep thermal decomposition at higher temperatures. TG and DTG curves (each *n* = 2) and corresponding mass spectra indicate polymer pyrolysis. In the helium atmosphere, prominent peaks at m/z 41, 69, 87, and 113 were consistently detected across all regions, indicating the presence of HEMA and EGDMA fragments and confirming the absence of CBD after post-processing. See also Figures S11—S16 for further information. (C) Load-displacement curves from quasi-static nanoindentation reflect microstructural heterogeneities (*n* = 9), as the observed variations indicate. Slight differences in elastic modulus and hardness are attributed to the templating process. Individual measurements are shown as data points, while lines demonstrate the average values.

The thermal performance of the 3D functional polymers was systematically evaluated for their suitability for real-world applications. Analysis using temperature-modulated differential scanning calorimetry (TMDSC) revealed a broad glass transition region ranging from approximately 100°C to about 180°C, with midpoint glass transition temperatures (*T*_g_) of 140.3 ± 0.1°C for 3DMIP and 140.9 ± 0.1°C for 3DNIP (see reversible heat flow in Figure 6A). Both materials remain in a rigid, glassy state at ambient conditions, which promotes stability during handling. The minimal change in reversible heat flow reflects the highly crosslinked nature of the polymer networks, which restricts segment mobility and supports structural integrity, even near *T*_g_ conditions. This thermal resilience permits operation at elevated temperatures during incubation or extraction without compromising structural integrity, facilitating enhanced diffusion and sorption kinetics, and potentially enabling efficient material regeneration. Beyond the glass transition, the polymers undergo thermal degradation, as evidenced by nonreversible heat flow signals.

Thermal degradation of 3DMIP was further analyzed via thermogravimetry mass spectrometry (TG-MS) and validated by thermal desorption gas chromatography-mass spectrometry (TD-GC/MS). Decomposition onset occurred around 180°C, followed by a multistep decomposition pattern (regions A–C), indicative of spatial variation in crosslinking density or chain microstructures (see TG and corresponding DTG curves in Figure 6B). The evolved gas analysis showed characteristic fragments of HEMA and EGDMA in all regions, demonstrating polymer backbone degradation. This finding was validated using TD-GC/MS, as shown in the supplemental information Figures S11–S15, and S16. No CBD was detected, demonstrating complete template removal after the post-processing procedure of the MIPs. These results highlight the robustness of the material under thermal stress and support its suitability for thermal exposure during regeneration.

Quasi-static nanoindentation under ambient conditions revealed average elastic moduli of 93 MPa (3DMIP) and 74 MPa (3DNIP) and hardness values of 5 MPa and 3 MPa, respectively, suggesting a slightly higher flexibility and malleability of the 3DNIP (Figure 6C). In general, both polymers are soft materials and are deformable. Variability within the data is attributed to topographical features (that is, surface roughness) and internal voids. The observed mechanical differences likely result from templating-induced microstructural changes and are in line with findings by Neusser et al., which highlight the dual chemical (e.g., functionality) and physical (e.g., mechanical) impact of molecular imprinting.^43^

Overall, our results demonstrate that combining CAD-defined macroarchitecture, hierarchical porosity, and molecular imprinting enables the fabrication of 3D functional polymers for specific CBD enrichment. This integration of precise structural design with molecular recognition capabilities directly into printable macroscopic geometries and the demonstrated transferability of the technology provide a versatile and scalable platform with high potential, e.g., not only for the purification of dietary supplements but also for the removal of contaminants in environmental applications. The thermal properties characterized here further support the suitability of these 3DMIPs for purification, sensing, and preconcentration applications.

### Limitations of the study

This study reports on the successful transfer of the 3DMIP fabrication workflow from an environmentally relevant hormone to the pharmaceutically promising CBD and presents the first comprehensive characterization of LCD-based 3D-printed, freestanding MIP monoliths. While these results are promising, several aspects require further investigation.

First, since this work focused on both in-depth material characterization to understand the system fundamentals and demonstrating transferability, the binding capacities and uptake kinetics of the 3DMIPs remain to be optimized. Future efforts should aim at tailoring the macrostructure and resin composition to fine-tune porosity and functionality. In this context, the printability of resins containing nonpolar or less polar porogens — promising for noncovalent imprinting and PIPS — should be investigated, as such formulations could reduce interference with template interactions and enable distinct porous morphologies. However, it should be noted that the binary solvent mixture used here creates monolithic separation media with meso- and macropores, the latter facilitating convective flow and efficient solute uptake. Alternative monomers, such as benzyl methacrylate for π-π interactions, could further enhance binding performance. Computational design approaches, including multi-monomer docking protocols, may support a more rational development of high-affinity 3DMIPs.^44^ In addition, future optimization studies should also include polymer swelling measurements to evaluate solvent-dependent structural stability and its influence on pore size and accessibility. Such data would further elucidate how diffusion and binding behavior are affected by environmental conditions. Complementary spectroscopic characterization, particularly Fourier transform infrared spectroscopy and nuclear magnetic resonance spectroscopy, could be valuable for optimizing the resin formulation and understanding structure-function relationships by confirming composition, assessing the conversion of crosslinker double bonds, and revealing structural differences between formulations.

Second, the binding experiments were conducted in model solutions with defined CBD concentrations in batch mode using freestanding lattice cubes. For practical large-scale CBD purification, future work should focus on integrating 3DMIPs into flow-through systems (e.g., as cartridge inserts for automated solid-phase extraction) and systematically assessing parameters such as fluid dynamics, pressure drop, reusability (including regeneration protocols), and long-term stability. Benchmarking with established CBD purification methods is essential for evaluating performance and scalability. These studies should be conducted in a concentration range likely to occur in the field, as the model concentration used in the batch-mode experiments does not fully reflect the broad dynamic range in real plant extracts. Additionally, to isolate the contribution of the 3D-printed macrostructure to binding performance, future studies should include simple solution-polymerized MIP monoliths as benchmarks. After controlled crushing and sieving the particles to match the wall thickness of the 3D-printed lattices, these could be evaluated in batch binding tests to provide insights into the intrinsic binding efficiency of the polymer formulations independent of their macroscopic design.

Third, the performance of 3DMIPs in complex plant extracts containing structurally related cannabinoids and matrix interferences remains to be validated. Competitive binding studies, for example, using high-performance liquid chromatography, are required to assess specificity under real-world conditions. Once 3DMIPs suitable for flow operation are established, integration with adaptable 3D-printed optical detectors, as proposed by Prado et al., could enable online absorbance monitoring.^45^

Despite these limitations, the presented results highlight the great potential of LCD-based 3D printing for molecular imprinting. These tailored polymers are promising for various applications — not only in separation, particularly for the purification and isolation of health-promoting compounds from complex plant extracts for nutraceuticals and medical research — but also in sensors, controlled release systems, and biomedical engineering.

Beyond its technical performance, the 3DMIP concept aligns with the principles of sustainable innovation. The LCD-based 3D printing process enables the zero-solid-waste, on-demand fabrication of specific adsorbents, thereby minimizing material waste. The expected reusability of the polymers and the low-energy enrichment process contribute to resource-efficient separations. With suitable modifications, the approach could also target other bioactive substances, such as polyphenols from grape seed extracts, facilitating the utilization of agricultural by-products and general waste from food processing and thus promoting sustainable resource use.

## Resource availability

### Lead contact

Requests for further information and resources should be directed to and will be fulfilled by the lead contact, Mehmet Dinc (mehmet.dinc@hahn-schickard.de).

### Materials availability

All unique/stable resins and polymers generated in this study are available from the lead contact with a completed materials transfer agreement.

### Data and code availability

• Data reported in this article will be shared by the lead contact upon request.
• This article does not report original code.
• Any additional information required to reanalyze the data reported in this study is available from the lead contact upon request.

## Acknowledgments

This work was financially supported by 10.13039/100017092Toray Research Center, Inc., Japan, and partially funded by ZIM (Zentrales Innovationsprogramm Mittelstand) under the project MIPextract (grant KK5054610SK1). B.K. wishes to thank the entire 10.13039/100017092Toray Research Center team in Otsu (Shiga, Japan) for their exceptional hospitality during his research stay and for the excellent cooperation. Special thanks goes to Kazutaka Tomita and Ryo Kuroda for their outstanding support during his stay.

## Author contributions

Conceptualization, B.K., Y.Y., T.Y., B.M., and M.D.; methodology, all authors; investigation, all authors except Y.Y., T.Y., B.M., and M.D.; formal analysis, B.K., A.H., H.S., Y.F., E.B.S.O., T.O., S.Y., and M.D.; writing — original draft, B.K., H.S., and Y.F.; writing — review & editing, all authors except B.K., H.S., and Y.F.; visualization, B.K., H.S., Y.F., E.B.S.O., F.U., and M.D.; funding acquisition, B.M. and M.D.; resources, T.Y., B.M., and M.D.; project administration, Y.Y., H.S., T.Y., and M.D.; supervision, T.Y., B.M., and M.D.

## Declaration of interests

B.K., B.M., and M.D. are co-inventors on a patent application related to the LCD-based 3D-printed MIP technology described in this manuscript (PCT/EP2024/076002). One of the authors of this study, B.M., is a guest editor of the special issue “Translational and interdisciplinary advances of molecular imprinting technology” to which this paper belongs.

## STAR★Methods

### Key resources table

**Table:** 

REAGENT or RESOURCE	SOURCE	IDENTIFIER
**Chemicals, peptides, and recombinant proteins**		

2-Hydroxyethyl methacrylate (HEMA)	Sigma Aldrich	CAS: 868-77-9
Ethylene glycol dimethylacrylate (EGDMA)	Sigma Aldrich	CAS: 97-90-5
Cyclohexanol	Thermo Fisher Scientific	CAS: 108-93-0
1-Decanol	Sigma Aldrich	CAS: 112-30-1
Bis(2,4,6-trimethylbenzoyl)-phenylphosphineoxide	S u. K Hock GmbH	CAS: 162881-26-7
Cannabidiol, crystalline >98%	Ai Lab Swiss AG	CAS: 13956-29-1
Progesterone, 98%	Thermo Fisher Scientific	CAS: 57-83-0
Quercetin dihydrate, 97%	Thermo Fisher Scientific	CAS: 6151-25-3
Ethanol absolute, LiChrosolv	Merck	CAS: 64-17-5
Methanol, EMSURE	Merck	CAS: 67-56-1
R-744 carbon dioxide 4.5, 99.995%	MTI	N/A

**Software and algorithms**		

OpenSCAD 2021.01	Open-source platform	https://openscad.org/
Chitubox V1.8.1	CBD Technology Ltd.	https://www.chitubox.com/en/index
Auto Slice & View 4.2	Thermo Fisher Scientific	https://www.thermofisher.com/order/catalog/product/de/de/autosliceview4
Avizo 3D 2023.2.	Thermo Fisher Scientific	https://www.thermofisher.com/de/de/home/electron-microscopy/products/software-em-3d-vis/avizo-software.html
Origin 2022b	OriginLab Corporation	https://www.originlab.com/

### Method details

#### Formulation of photocurable resins for 3DMIP and 3DNIP fabrication

To ensure comparability, both 3DMIPs and their nonimprinted counterparts (control polymers; 3DNIPs) were fabricated from identical photocurable resin formulations and identical printing parameters and post-treatment conditions. In the case of 3DNIPs, the template molecule – CBD – was omitted.

The resins were freshly prepared in amber glass vials by combining 26 wt% HEMA, 23 wt% EGDMA, 9.5 wt% 1-decanol, 39.5 wt% cyclohexanol, and 2 wt% BAPO as photoinitiator. The mixture was homogenized by manual stirring followed by 15 min of sonication. The resulting resin appeared clear and yellowish and was stored in the dark at 4 °C to prevent prepolymerization.

The porogen system, consisting of 1-decanol and cyclohexanol, was chosen due to its compatibility with PIPS and noncovalent imprinting in LCD-based 3D printing, enabling the efficient formation of porous polymer networks.

For 3DMIP synthesis, 1.52 mmol CBD was dissolved in 40 mL of resin, yielding a clear and light green solution. Prior to printing, the mixture was sonicated for 10 min at 23 °C to facilitate the noncovalent self-assembly of the template-monomer complex, which is crucial for generating recognition sites during polymerization.

#### Design and slicing of 3D lattice models

Two 3D lattice architectures were designed using the script-based modeling tool OpenSCAD to investigate the influence of macrogeometry on adsorption performance. The first design (Shape 1, S1) comprised a fine lattice cube (9.5 × 9.5 × 9.5 mm^3^) with 8 × 8 square windows per face, each measuring 760 × 760 μm^2^, and a wall thickness of 380 μm. The second design (Shape 2, S2) featured a coarser lattice cube (9 × 9 × 9 mm^3^) with 3 × 3 windows per face (1.5 × 1.5 mm^2^) and a set wall thickness of 1.5 mm.

The corresponding 3D models were exported as STL files using custom OpenSCAD scripts. For printing, the STL files were imported into the CHITUBOX slicer software and digitally sliced into 2D horizontal layers with a thickness of 50 μm.

Script for generating the S1 lattice cube

a=9.5; //size of the cube in mm

l = 0.76;//lattice spacing in mm

g = 0.38;//lattice walls in mm

b = 0.5;//correction value.

$fn = 100;

t = g+l;

z = -b+round((0.5∗((a)/t)));

latticed_cube();

module latticed_cube()

{difference(){

cube(size=a, center = true);

lattice();}}

module lattice(){

y_baselattice();

x_baselattice();

z_baselattice();}

module x_basegrid()

{for(c=[-z:z]) translate([(t∗c), 0, 0]) cube([l,a,l], center = true);}

module y_basegrid()

{for(c=[-z:z]) translate([0, (t∗c), 0]) cube([a,l,l], center = true);}

module z_basegrid()

{ for(c=[-z:z]) translate([(t∗c), 0, 0]) cube([l,l,a], center = true);}

module x_baselattice()

{for(c=[-z:z]) translate([0, 0, (t∗c)]) x_basegrid();}

module y_baselattice()

{for(c=[-z:z]) translate([0,0, (t∗c)]) y_basegrid();}

module z_baselattice()

{for(c=[-z:z]) translate([0,(t∗c), 0]) z_basegrid();}

Script for generating the S2 lattice cube

a=9.0; //size of the cube in mm

l=1.5; //lattice spacing in mm

g=1.5; //lattice walls in mm

$fn=100;

t=g+l;

z=floor((a - t) / (2 ∗ t));

latticed_cube();

module latticed_cube()

{difference(){

cube(size=a, center = true);

lattice();}}

module lattice(){

y_baselattice();

x_baselattice();

z_baselattice();}

module x_basegrid()

{for(c=[-z:z]) translate([(t∗c), 0, 0]) cube([l,a,l], center = true);}

module y_basegrid()

{for(c=[-z:z]) translate([0, (t∗c), 0]) cube([a,l,l], center = true);}

module z_basegrid()

{ for(c=[-z:z]) translate([(t∗c), 0, 0]) cube([l,l,a], center = true);}

module x_baselattice()

{for(c=[-z:z]) translate([0, 0, (t∗c)]) x_basegrid();}

module y_baselattice()

{for(c=[-z:z]) translate([0,0, (t∗c)]) y_basegrid();}

module z_baselattice()

{for(c=[-z:z]) translate([0,(t∗c), 0]) z_basegrid();}

#### Fabrication of 3DMIPs and 3DNIPs via LCD-based 3D printing

All 3DMIPs and 3DNIPs were created using a commercially available, low-cost LCD-based 3D printer (Phrozen Sonic Mini 4K, Phrozen Technology) equipped with a 405 nm UV LED matrix that matches the absorption spectrum of BAPO. For each printing batch, 40 mL of the respective resin was dispensed into the printer vat. The printing parameters were set as follows: (bottom) exposure time of 19 s, (bottom) light-off delay of 9 s, (bottom) lifting speed of 80 mm/min, and lifting distance of 5 mm. A flexible spring steel plate was magnetically attached to the build platform to facilitate the detachment of the printed structures.

#### Post-processing and template removal via solvent washing and supercritical carbon dioxide drying

A sequential washing protocol enabled the removal of non-polymerized components – including unreacted monomers, photoinitiator, and porogens – from the printed 3D structures: two rinses with technical-grade acetone, immersion in technical-grade isopropanol for 150 min, followed by two washes with absolute ethanol. For 3DMIPs, this procedure also enabled template extraction. All samples were stored in absolute ethanol until subjected to supercritical drying.

To prevent structural deformation and preserve the pore architecture, the samples were supercritically dried (CPD300, Leica Microsystems). The samples were placed in fresh absolute ethanol. After the gradual replacement of ethanol with liquid carbon dioxide, supercritical conditions (35 °C; 79 bar) were established. Controlled depressurization of the chamber yielded gently dried polymers. Beyond structural integrity, the supercritical carbon dioxide environment enhances the removal of residual template molecules, thereby minimizing the risk of analyte leaching in subsequent applications.

#### Batch incubation binding studies and adsorption behavior of 3DMIP monoliths

Batch adsorption experiments were carried out in triplicate (*n* = 3). Polymer monoliths were incubated in 100 mL of a 3:1 (v/v) water:methanol solution containing 24 μmol/L CBD for 60 min at ambient temperature on a rocking platform (120 rpm). All experiments were conducted under identical conditions, unless otherwise noted, to ensure comparability of adsorption capacities.

Following incubation, the monoliths were transferred to 3 mL of absolute ethanol and gently agitated for 15 min (120 rpm; rocking platform) to extract the bound analyte. CBD concentrations in the supernatants were quantified by UV/VIS spectroscopy at 280 nm using a quartz cuvette with a 1 cm path length (Specord S600, Analytik Jena). Each sample was analyzed in duplicate, with five replicates per measurement.

The enrichment factor represents the ratio of the eluted CBD concentration to the original incubation concentration. To elucidate geometry-dependent effects on the binding behavior of dried 3DMIPs and 3DNIPs, polymers of both geometries (S1 vs. S2) were employed. Time-dependent studies were conducted with S1-type polymers at incubation times of 15, 30, and 60 min, as well as 4 and 21 hours.

The pH dependency of adsorption was assessed by determining the adsorption capacity after incubation of S1-type polymers in 100 mL of a 3:1 (v/v) MeOH:phosphate-buffered saline (PBS) solution (containing 24 μmol/L CBD) at pH values of 3.8, 7.0, and 11.8, followed by extraction as described above.

For binding studies with quercetin and progesterone, S1-type polymers were incubated in 100 mL of a 3:1 (v/v) water:methanol solution containing 24 μmol/L quercetin or progesterone. Absorbance measurements were performed at 375 nm (quercetin) and 240 nm (progesterone).

#### Surface area and pore size distribution by nitrogen sorption

Nitrogen sorption isotherms of the supercritically dried polymer samples (S1 3DMIP, S1 3DNIP, S2 3DMIP, S2 3DNIP) were acquired using a BELSORP 18 instrument (MicrotracBEL). Prior to analysis, samples were mechanically crushed. All measurements were conducted at 77 K with an equilibrium time of 180 s per point. For pretreatment, samples were degassed under vacuum at 50 °C for 24 hours. Based on the adsorption data, specific surface areas were calculated according to the BET method, while pore size distributions were obtained by GCMC simulations.

#### Determination of total porosity by helium pycnometry and mercury intrusion porosimetry

Mercury intrusion porosimetry (AutoPore V 9620, Micromeritics) and helium pycnometry (AccuPyc II 1340, Micromeritics) were employed to determine the pore size distribution, skeletal density, and total porosity of supercritically dried S1-type polymers. For mercury porosimetry, intact monoliths were degassed under vacuum prior to analysis. The measurement range covered pore diameters from approximately 3 nm to 400 μm. Specific pore volumes and pore size distributions were derived from intrusion data using the Washburn equation, assuming a contact angle of 130° and a surface tension of 0.484 N/m.

Helium pycnometry was performed at 25 °C on mechanically crushed polymers. Each sample was analyzed with five replicates per measurement. The skeletal density was calculated from the ratio of sample mass to measured volume. Five measurements were performed for each sample.

#### FIB/SEM tomography for 3D morphological characterization

FIB/SEM tomography was employed to visualize and quantify the inherent porosity and nanoscale architecture of the 3D-printed soft materials. Sample preparation was adapted from a previously reported protocol for hierarchically porous, methacrylate-based polymers.^46^ Briefly, core fragments (i.e., pieces from the center of a pillar) of S2 3DMIP and 3DNIP cubes were embedded in silicone resin via vacuum assistance to ensure pore filling and sufficient contrast. After curing, the samples were further embedded in epoxy resin to facilitate handling. The resulting blocks were ground with a file and smoothed with a single-edged blade to expose the polymer structures, followed by sputter-coating with an approximately 10 nm platinum layer (JEC-3000FC, JEOL) to minimize charging during FIB/SEM investigation.

Automated FIB/SEM tomography was conducted on a Helios G4 DualBeam system (Thermo Fisher Scientific) equipped with Auto Slice & View 4.2 software. Serial cross-sections were milled with a 30 kV Ga^+^ at 83 pA, while backscattered electron (BSE) images were acquired at 1 kV and 0.17 nA using a through-the-lens detector (TLD). The analyzed and reconstructed volumes measured 3.6 × 3.2 × 1.2 μm^3^ for 3DMIP and 3.3 × 3.4 × 1.2 μm^3^ for 3DNIP, with a voxel size of 2.7 × 3.4 × 3.0 nm^3^.

This high-resolution tomographic approach enabled the detailed reconstruction of the highly structured polymer network and provided quantitative insights into porosity, pore interconnectivity, and pore size distribution, which are key parameters in understanding and optimizing 3DMIPs.

#### Image stack processing and local thickness analysis

The electron image stacks were processed using Avizo 3D to reconstruct and quantify the internal porous architecture. After the alignment of the BSE images, noise reduction was performed using non-local means filtering. Binarization was achieved via the auto thresholding function (threshold levels: 97 for 3DMIP, 98 for 3DNIP). Subsequently, morphological smoothing was applied using the smooth labels function to refine pore–polymer interfaces. The processed image stacks were then segmented and rendered in 3D for visualization of the internal structures.

#### Surface composition via TOF-SIMS investigation

TOF-SIMS analyses were performed to compare the surface composition of dried S2-type 3DMIP and 3DNIP samples and to obtain cross-sectional images (TOF.SIMS 5, IONTOF). Positive and negative ion spectra were acquired using a Bi_3_^2+^ primary ion source over a raster area of 200 μm^2^. For the cross-sectional images in positive ion mode, samples were embedded in epoxy resin and cryosectioned using a Leica EM UC7 equipped with an EM FC7 cryochamber.

#### Thermal properties assessed by TMDSC

TMDSC was performed using a Q1000 calorimeter (TA Instruments) to analyze the thermal behavior of dried 3DMIPs and 3DNIPs (approximately 5 mg, measured in duplicate). Dry nitrogen gas was purged through the cell at a flow rate of 50 mL/min. The experiments were conducted with a linear heating rate of 2 K/min and a sinusoidal temperature modulation (amplitude: ±0.318 K; period: 60 s). Reversible and non-reversible heat flow components were extracted from the modulated signal using the instrument software. The measurement was performed in duplicate.

#### Thermal decomposition analysis using TG-MS and TD-GC/MS

The thermal degradation behavior of dried S1-type 3DMIP samples (approximately 1 mg) was investigated by TG-MS. The thermogravimetric measurements were performed in a helium atmosphere, heating the samples from 30 °C to 700 °C at a constant heating rate of 10 °C/min (STA 2500 Regulus, NETZSCH). Evolved gases were analyzed *in situ* using a quadrupole mass spectrometer directly coupled to the TG system (QP-2020SE, Shimadzu). The measurement was performed in duplicate.

To validate and further elucidate the pyrolysis products, TD-GC/MS was employed. The volatile fractions were collected in three defined temperature ranges: region A (30–280 °C), region B (280–400 °C), and region C (400–700 °C), each with a heating rate of 10 °C/min. After a flush time of 5 min, evolved gaseous products were adsorbed onto Tenax tubes with a nitrogen flow rate of 50 mL/min. The tubes were then desorbed at 260 °C for 15 min in a two-stage tube desorption mode using helium as the carrier gas (flow rate: 50 mL/min) (TD100-xr, Markes International), and analyzed using GC/MS (GC7890/5975C, Agilent). Separation was achieved on a DB-5MS column (30 m × 0.25 mm × 1 μm) under the following temperature program: 40 °C (4 min hold), ramped at 8 °C/min to 280 °C (held for 16 min).

#### Mechanical characterization by nanoindentation

Quasi-static nanoindentation experiments were conducted under ambient conditions using a TriboIndenter Hysitron TI950 (Bruker) equipped with a Berkovich diamond tip to assess the mechanical properties of solid, inherently porous 3DMIP and 3DNIP monoliths (5 × 5 × 5 mm^3^). The nanoindentation tests were performed at the center of each sample surface. The maximum load was set to 400 μN, with loading, holding, and unloading times of 5 s. Nine measurements were performed for each sample.

### QUANTIFICATION AND STATISTICAL ANALYSIS

The diagrams presented in this paper were generated from the original data using Origin 2022b.

#### Batch incubation binding studies and adsorption behavior of 3DMIP monoliths

Quantification was based on a linear seven-point calibration function (0.10–0.50 mmol/L) established in ethanol. The adsorption capacity (*Q*), defined as the amount of adsorbed target analyte per gram of polymer, was calculated according to Equation 1:

(Equation 1) $$Q=\frac{C_{s}\;V_{s}\;M_{CBD}}{m_{P}}$$

where *C*_*s*_ is the molar concentration of eluted CBD, *V*_*s*_ is the extraction volume (3 mL), *M*_*CBD*_ is the molar mass of CBD, and *m*_*p*_ is the mass of the 3D polymer. The imprinting factor (*IF*), which reflects the specific recognition capability of the 3DMIPs, was calculated using Equation 2:

(Equation 2) $$IF=\frac{Q_{3DMIP}}{Q_{3DNIP}}$$

where *Q*_*3DMIP*_ and *Q*_*3DNIP*_ are the respective binding capacities of the polymers.

The adsorption kinetics were modeled using pseudo-first-order and pseudo-second-order models, described by Equations 3 and 4, respectively.

(Equation 3) $$Q_{t}=Q_{e}\;\left(1-e^{\left(-k_{1}\;t\right)}\right)$$

(Equation 4) $$Q_{t}=\frac{Q_{e}^{2}{\;k}_{2}\;t}{1+Q_{e}\;k_{2}\;t}$$

Here, *Q*_*t*_ and *Q*_*e*_ represent the adsorption capacities at time *t* and equilibrium, while *k*_*1*_ and *k*_*2*_ denote the rate constants for the respective kinetic models.

In addition to empirical kinetic models, diffusion-controlled adsorption was further analyzed for the S1-type polymers using Crank’s solution for diffusion into a plane sheet from a stirred solution of limited volume as a simplified approximation. The lattice cube was considered as a stack of lattice walls. The characteristic length was defined as half the thickness of a cube pillar (*l* = 190 μm), i.e., half the thickness of a lattice wall. The time-dependent fractional uptake *Q*_*t*_*/Q*_*∞*_ was fitted using Equation 5.^40^ Since *α*, the ratio of the analyte mass in solution to that in the polymer at equilibrium, is approximately 0.4402, Equation 5 is appropriate for modeling diffusion in a finite volume system such as the experimental setup described. For numerical evaluation of the apparent diffusion coefficients, the infinite series was approximated using the first six terms.

(Equation 5) $$\frac{Q_{t}}{Q_{\infty }}=1-\sum_{n=1}^{\infty }\frac{2\alpha \;\left(1+\alpha \right)}{{1+\alpha +\alpha }^{2}{q_{n}}^{2}}e^{\left(-\frac{D\;{q_{n}}^{2}t}{l^{2}}\right)}$$

Here, *Q*_*t*_ and *Q*_*∞*_ are the adsorption capacities at time *t* and equilibrium (approximated at 1260 min), *D* is the apparent diffusion coefficient, and *q*_*n*_ are values that can be interpolated from standard tables based on the *α* value.^40^ For *α* = 0.4402, the corresponding *q*_*n*_ values were determined as follows: *q*_*1*_ = 2.3435, *q*_*2*_ = 5.1320, *q*_*3*_ = 8.1.288, *q*_*4*_ = 11.1975, *q*_*5*_ = 14.2962, *q*_*6*_ = 17.4096.

For binding studies with quercetin and progesterone, quantification was based on linear calibration functions ranging from 0.01-0.06 mmol/L and 0.01-0.08 mmol/L, respectively.

#### Determination of total porosity by helium pycnometry and mercury intrusion porosimetry

Total porosity was calculated from the specific pore volume and the skeletal density according to Equation 6.

(Equation 6) $$\text{Porosity}\;\left[\%\right]=\frac{specific\;pore\;volume}{\left(\text{specific}\;\text{pore}\;\text{volume}+\frac{1}{skeletal\;density}\right)}*100\%$$

#### Image stack processing and local thickness analysis

For the determination of pore size distribution, quantitative local thickness analysis of the segmented data was performed based on a sphere-fitting algorithm (i.e., thickness map function), which calculates the diameter of the largest sphere that can be inscribed.^47^

#### Mechanical characterization by nanoindentation

The reduced modulus and hardness were extracted from the slope of the unloading curve using the Oliver–Pharr method. The elastic modulus was subsequently calculated according to Equation 7.

(Equation 7) $$E_{s}=\frac{1-v_{s}^{2}}{\left(\frac{1}{E_{r}}-\frac{1-v_{i}^{2}}{E_{i}}\right)}$$

Here, *E*_*s*_ is the elastic modulus of the sample; *E*_*r*_ is the reduced elastic modulus obtained from the indentation data; *E*_*i*_ is the elastic modulus of the indenter probe; *v*_*s*_ is the Poisson’s ratio of the sample (assumed to be 0.4 for the copolymer); and *v*_*i*_ is the Poisson’s ratio of the indenter probe.
