# Supplementary material for: From pixels to pores: 3D-(im)printed hierarchically porous polymer monoliths
Source: iScience. 2025 Dec 24;29(1):114539. doi: 10.1016/j.isci.2025.114539 (PMC12808914; doi:10.1016/j.isci.2025.114539)
Supplement: Document S1. Figures S1–S16 [file mmc1.pdf]

iScience, Volume *29*

Supplemental information

From pixels to pores: 3D-(im)printed hierarchically porous polymer monoliths

Benedikt Keitel, Simon Schimana, Amelie Huber, Yuki Yoshida, Hirotaka Shioji, Yoshitomo Furushima, Emine Billur Sevinis Ozbulut, Tomohiro Ohkawa, Shigeru Yoshimoto, Takashi Kubo, Hiroyuki Hosomi, Tsuyoshi Kato, Fumiya Uehara, Hiroko Futamura, Kana Nakanishi, Asuka Noda, Takashi Yamamoto, Boris Mizaikoff, and Mehmet Dinc

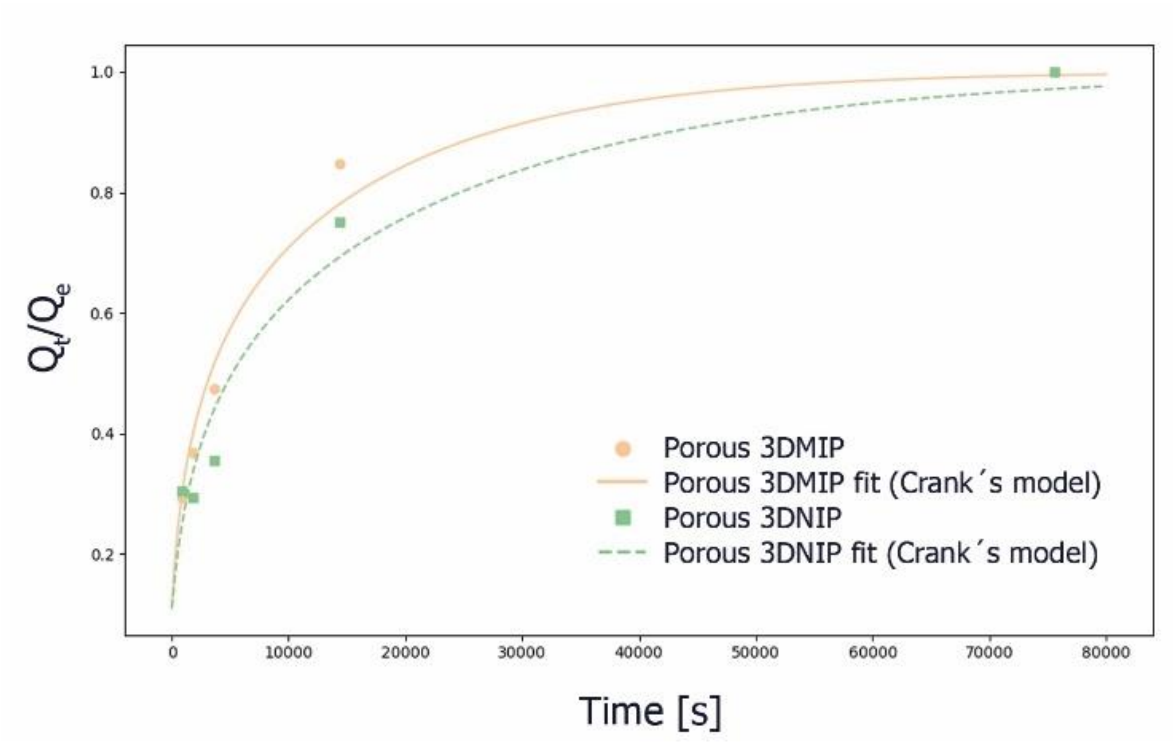

Figure S1. Fractional CBD uptake over time with experimental adsorption kinetics (symbols) and corresponding Crank's diffusion model fits (lines) for porous 3DMIPs and 3DNIPs (R^2 = 0.981 and RMSE = 0.038 for 3DMIPs and R^2 = 0.962 and RMSE = 0.056 for 3DNIPs), related to the adsorption kinetics paragraph of the Results and Discussion section. Crank's diffusion model for adsorption from a stirred finite–volume system into a planar sheet was applied to estimate the approximate apparent diffusion coefficients. The 3DMIP exhibited a higher apparent diffusion coefficient (3.89×10^{-13} m²/s) compared to the 3DNIP (2.50×10^{-13} m²/s), indicating facilitated mass transport in the imprinted material.

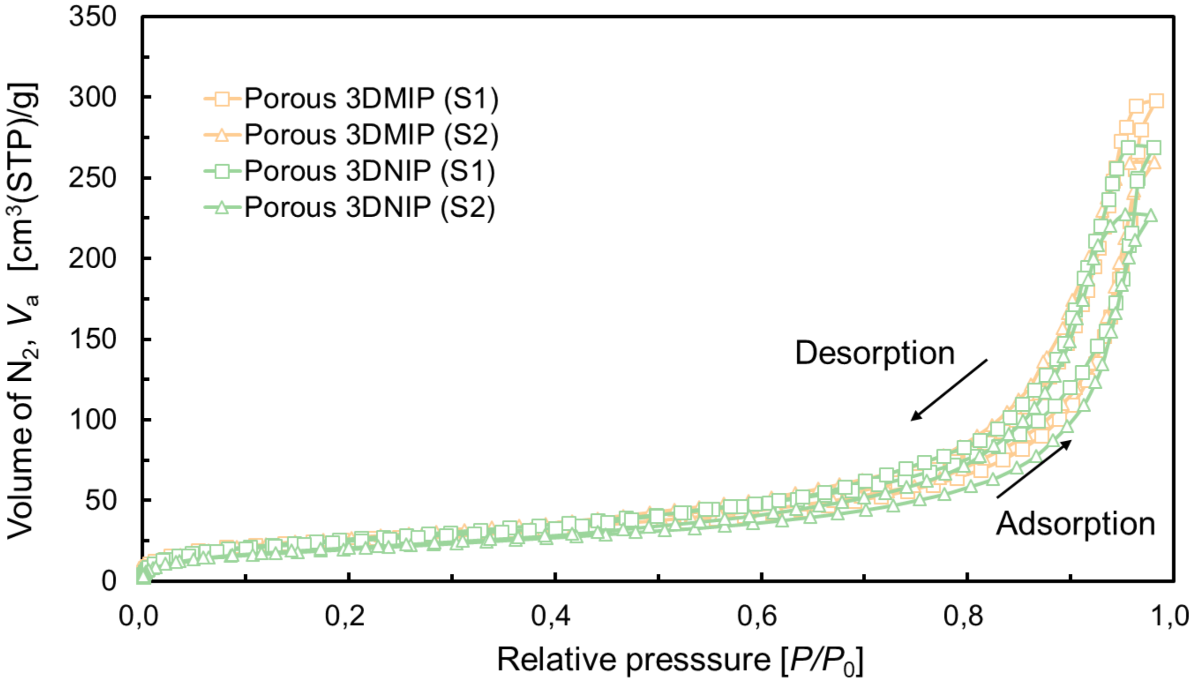

Figure S2. Nitrogen adsorption–desorption isotherms of 3DMIPs and 3DNIPs (S1 and S2 geometries) obtained from nitrogen sorption measurements, related to Table 1. All samples exhibit Type IV isotherms with H1-type hysteresis, characteristic of mesoporous materials with interconnected pore networks.

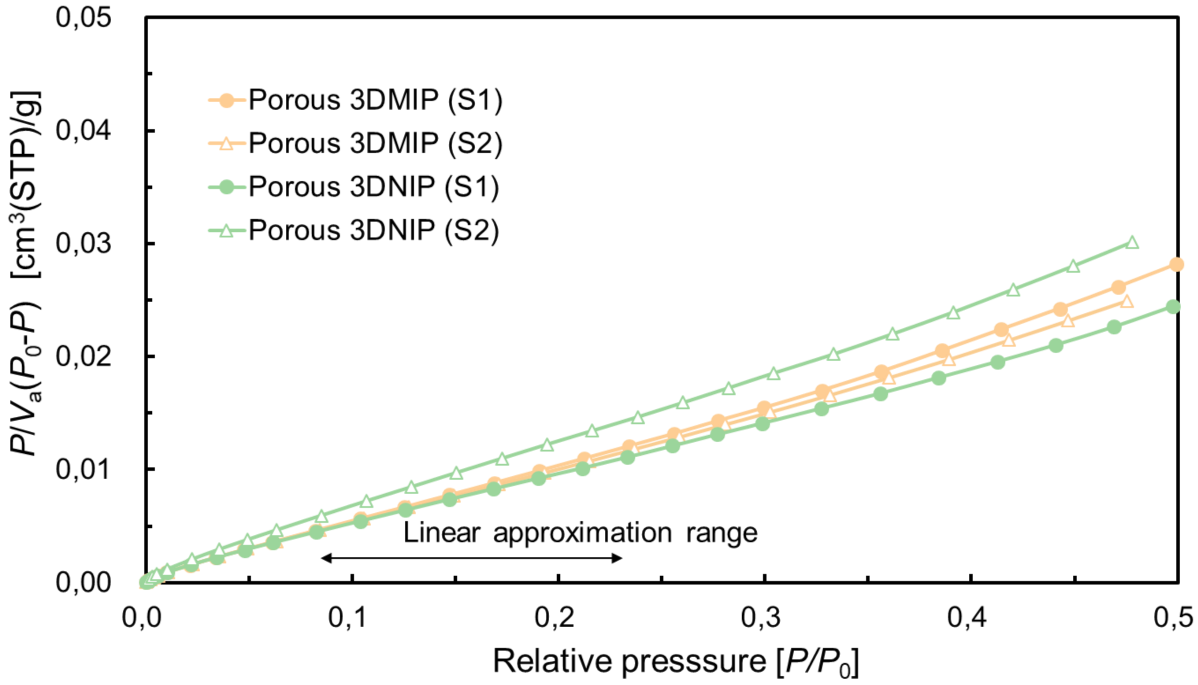

Figure S3. Brunauer–Emmett–Teller (BET) plot of nitrogen adsorption data, related to Table 1. The linear region in the selected relative pressure range confirms the applicability of the BET model for these samples.

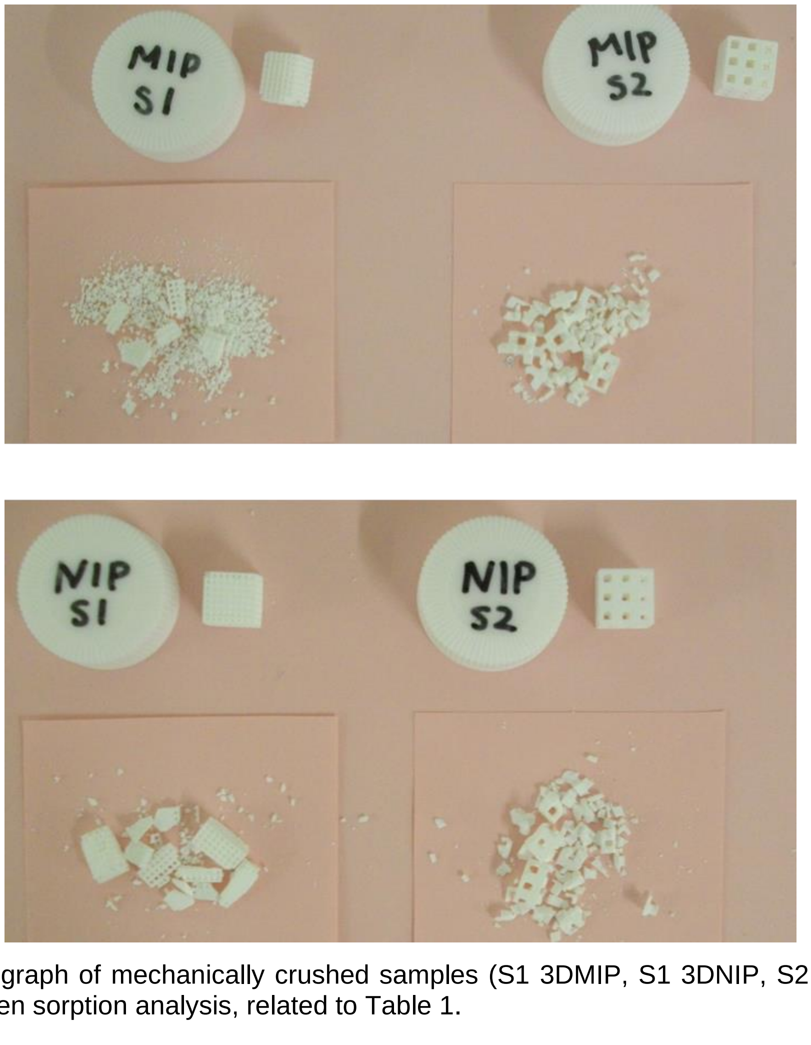

Figure S4. Photograph of mechanically crushed samples (S1 3DMIP, S1 3DNIP, S2 3DMIP, and S2 3DNIP) for nitrogen sorption analysis, related to Table 1.

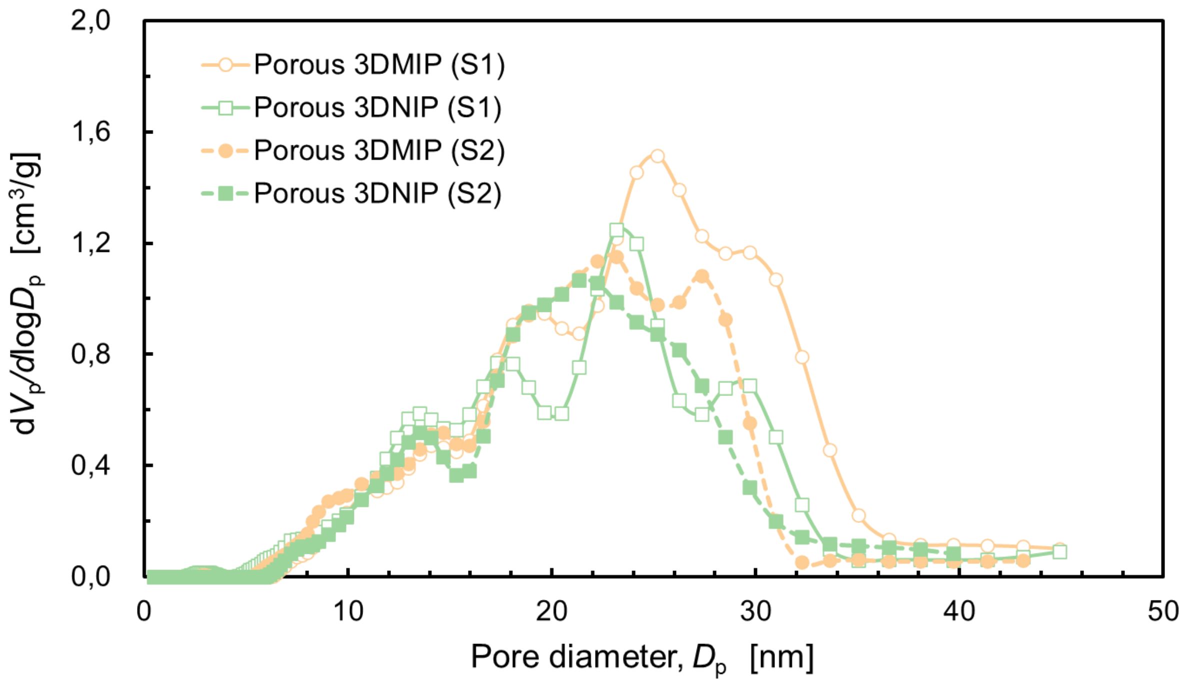

Figure S5. Pore size distribution derived from nitrogen adsorption using Grand Canonical Monte Carlo (GCMC) simulations, related to Figure 4B. The GCMC-derived mesopore distribution profile resembles the mesopore regime obtained by mercury intrusion porosimetry, showing a consistent trend: 3DNIPs tend to have slightly smaller pores than 3DMIPs.

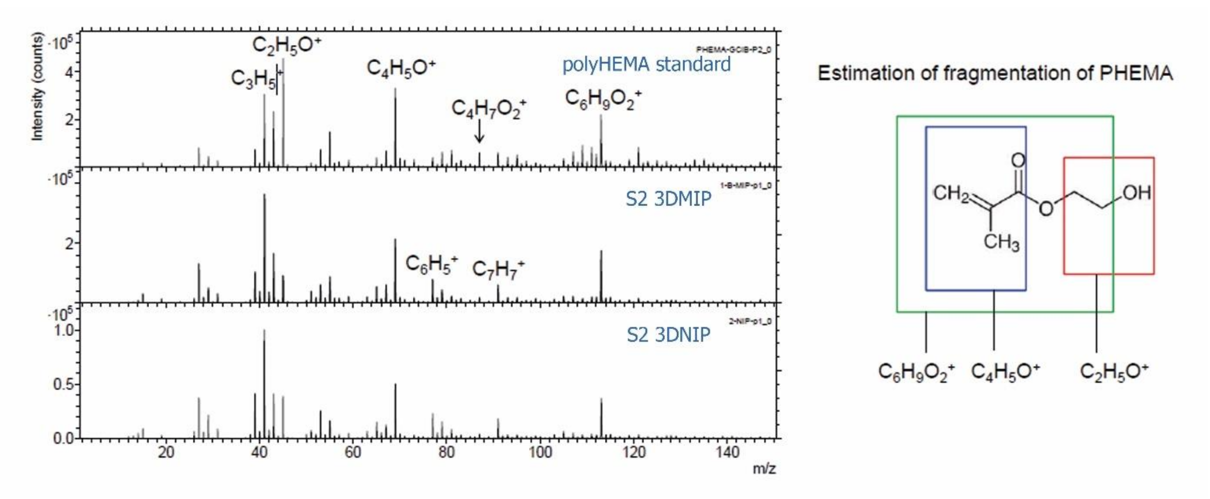

Figure S6. Positive ion spectra of S2 3DMIP and 3DNIP, obtained by time-of-flight secondary ion mass spectrometry (TOF-SIMS) analysis, show highly similar profiles, related to the TOF-SIMS paragraph of the Results and Discussion section. The detected ions are consistent with a polyHEMA standard. Differences in peak ratios compared to the standard (e.g., $C_2H_5O^+/C_4H_5O^+$) may arise from the contributions of both HEMA and EGDMA.

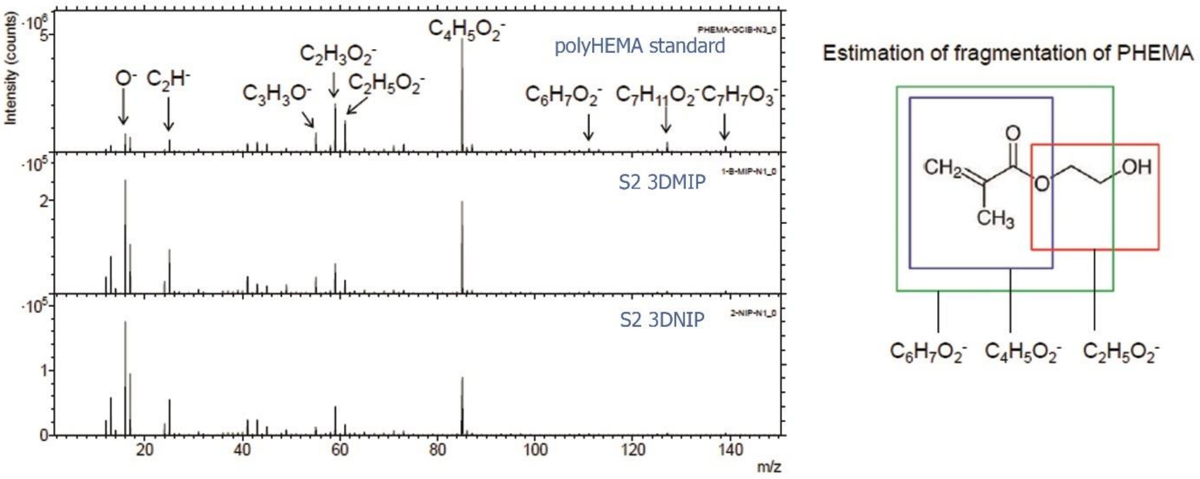

Figure S7. Negative ion spectra of S2 3DMIP and 3DNIP, obtained by TOF-SIMS analysis, exhibit highly similar profiles, related to the TOF-SIMS paragraph of the Results and Discussion section. The observed ions align with the polyHEMA standard. Similar ions are likely to be observed for both HEMA and EGDMA, with possible differences in peak ratios compared to the standard (e.g., $C_2H_5O_2^-/C_4H_5O_2^-$).

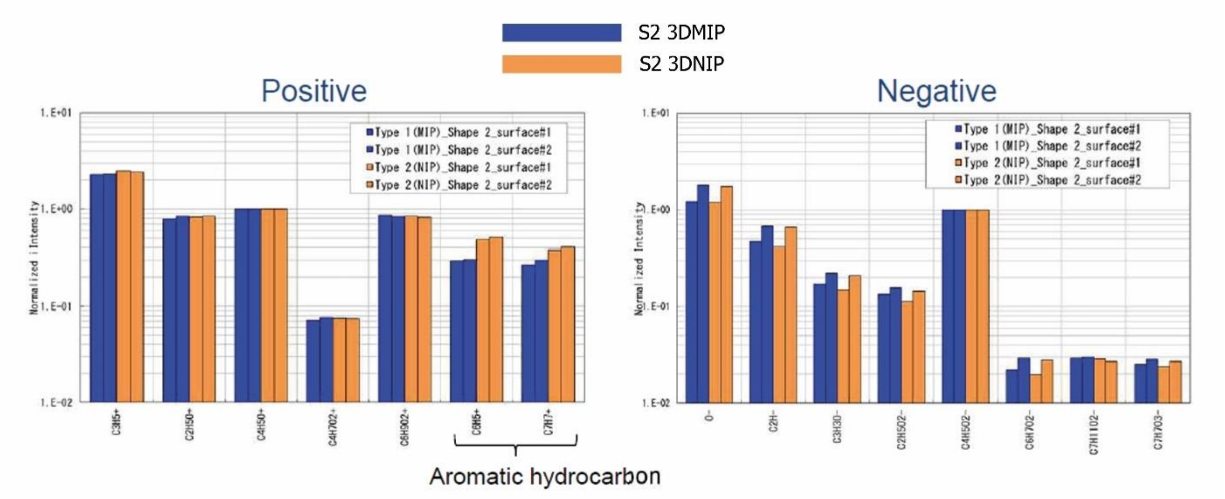

Figure S8. Comparison of TOF-SIMS peak intensities for S2 3DMIP and 3DNIP, normalized to the methacrylate main chain ($C_4H_5O^+$ or $C_4H_5O_2^-$), related to the TOF-SIMS paragraph of the Results and Discussion section. Positive and negative ion spectra show nearly identical profiles, with no significant differences in surface composition.

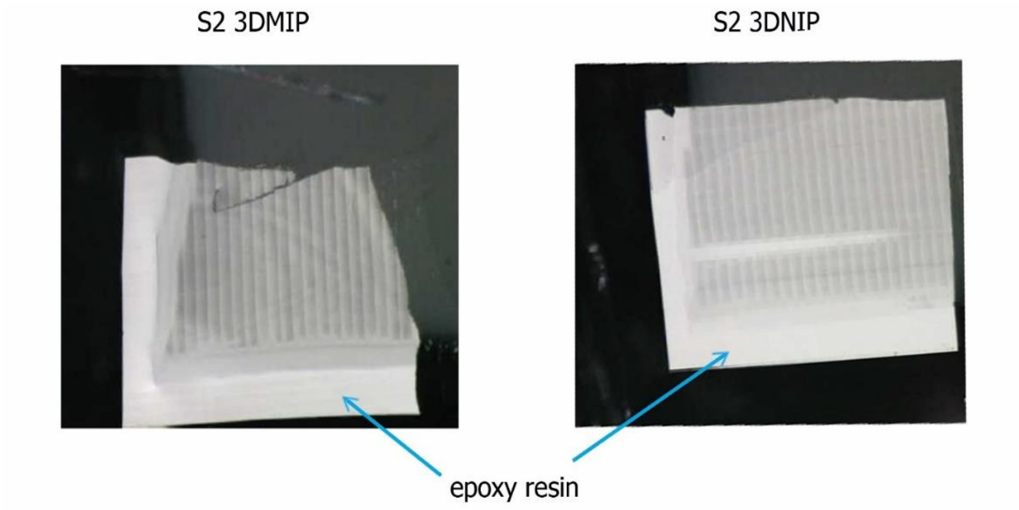

Figure S9. Light microscopy images of cryo-microtomed cross-sections of epoxy-embedded S2 3DMIP and 3DNIP samples prepared for TOF-SIMS positive ion imaging, related to the TOF-SIMS paragraph of the Results and Discussion section.

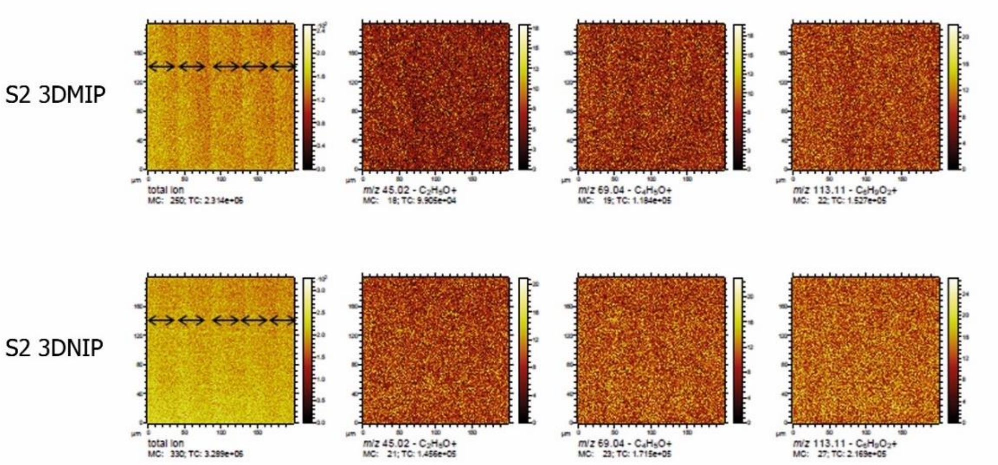

Figure S10. Positive ion TOF-SIMS images of cryo-microtomed cross-sections of epoxy-embedded S2 3DMIP and 3DNIP samples, related to the TOF-SIMS paragraph of the Results and Discussion section. The layered appearance in the total ion images results from surface roughness.

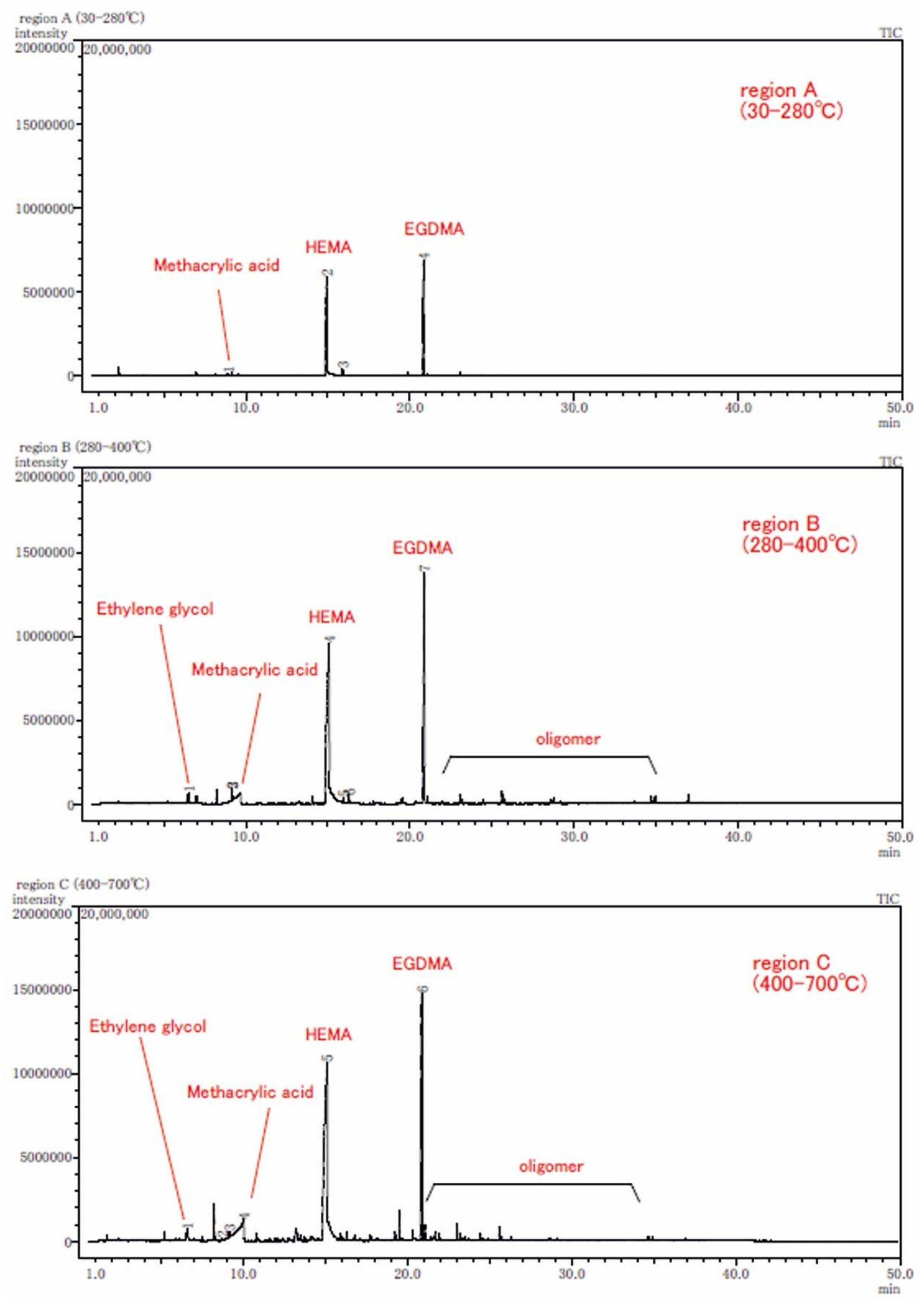

Figure S11. Total ion current chromatograms of outgassing from regions A (30–280 °C), B (280–400 °C), and C (400–700 °C) of S1 3DMIP obtained by thermal desorption gas chromatography-mass spectrometry (TD-GC/MS), related to the thermal degradation of 3DMIP paragraph of the Results and Discussion section.

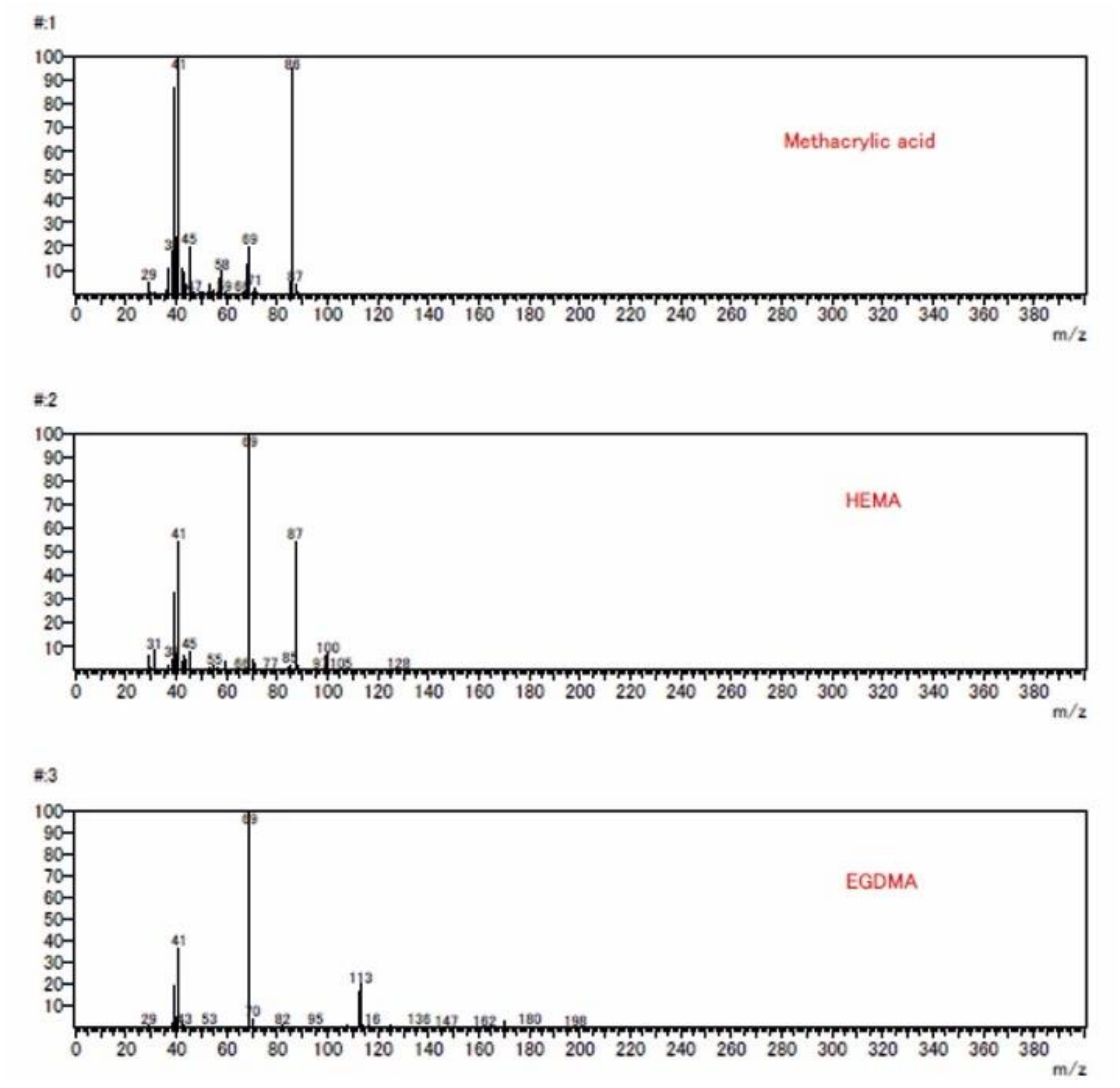

Figure S12. Mass spectra corresponding to outgassing from region A (30–280 °C) of S1 3DMIP obtained by TD-GC/MS, related to the thermal degradation of 3DMIP paragraph of the Results and Discussion section.

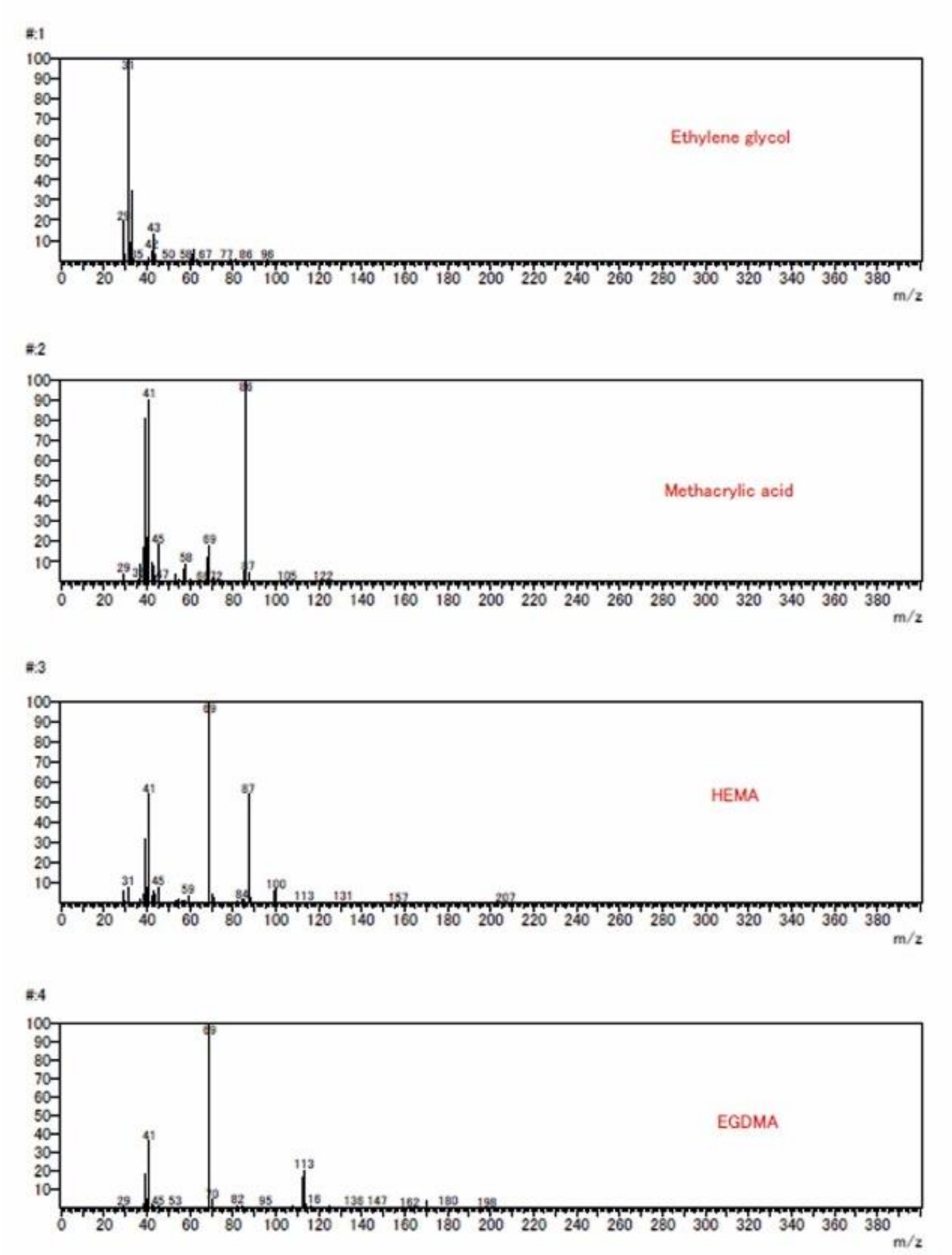

Figure S13. Mass spectra corresponding to outgassing from region B (280–400 °C) of S1 3DMIP obtained by TD-GC/MS, related to the thermal degradation of 3DMIP paragraph of the Results and Discussion section.

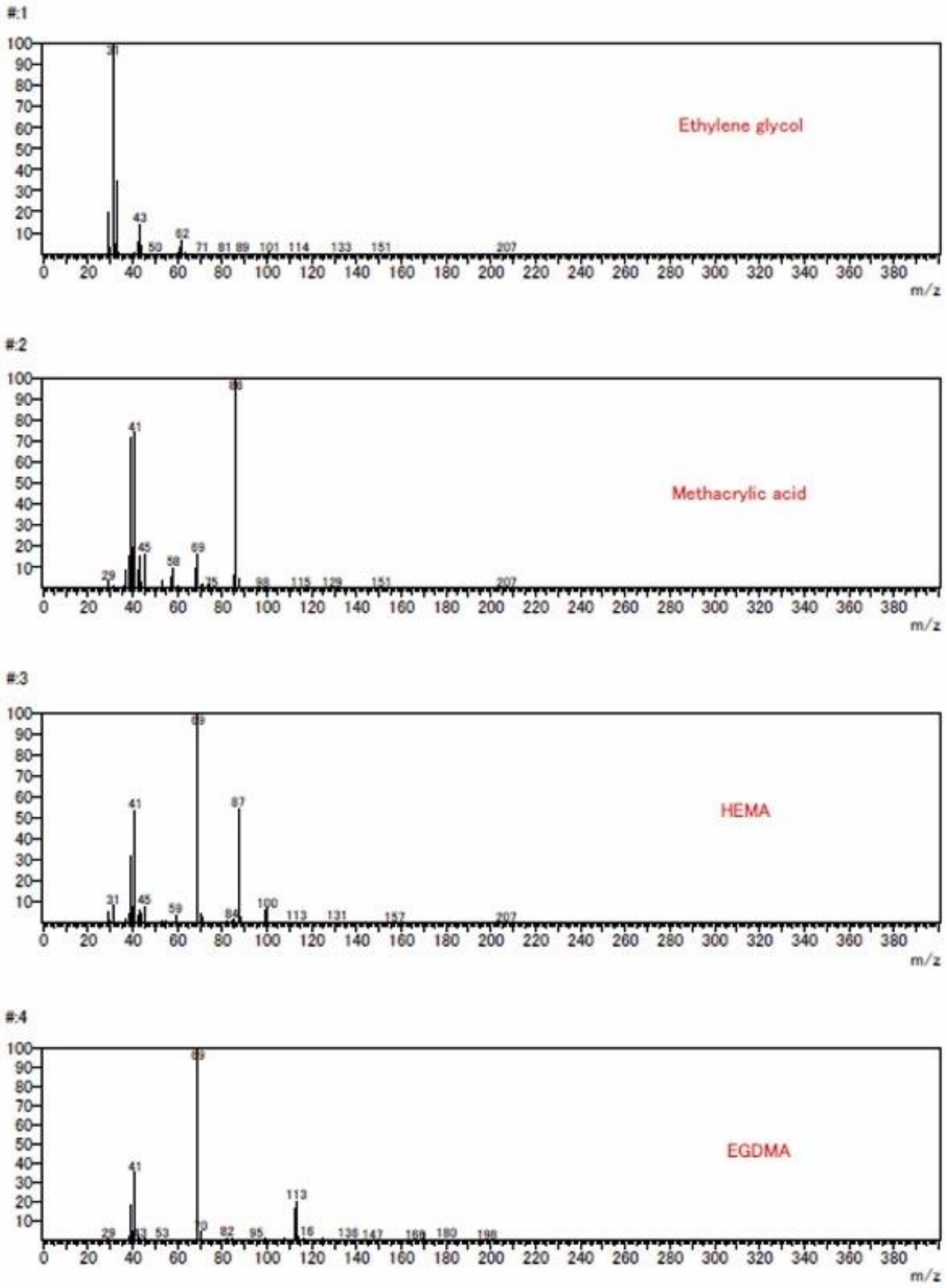

Figure S14. Mass spectra corresponding to outgassing from region C (400–700 °C) of S1 3DMIP obtained by TD-GC/MS, related to the thermal degradation of 3DMIP paragraph of the Results and Discussion section.

Table 2　　Region A：Analysis results of TD-GC/MS

Peak#	R.T. (min)	Compound	Sample mass (g)	Peak area	Detection amount (μg)	Amount generated from the sample (mass%)*1
1	9.0	Methacrylic acid	0.0107	1,177,469	3.776	0.035
2	14.9	2-Hydroxyethyl methacrylate (HEMA)	0.0107	35,515,729	113.899	1.1
3	14.9	Oxygen contained compound	0.0107	986,945	3.165	0.030
4	20.8	Ethylene glycol dimethacrylate (EGDMA)	0.0107	16,449,709	52.754	0.49
Total			0.0107	62,357,238	199.980	1.9

*1　Concentrations of VOCs were estimated based on Toluene response factor. Compounds out of calibration curve is calculated by extrapolation.

Table 3　　Region B：Analysis results of TD-GC/MS

Peak#	R.T. (min)	Compound	Sample mass (g)	Peak area	Detection amount (μg)	Amount generated from the sample (mass%)*1
1	6.5	Ethylene glycol	0.0107	5,176,413	16.601	0.16
2	9.2	Methacrylic acid	0.0107	16,892,482	54.174	0.51
3	9.2	Ethyl methacrylate + C7H14	0.0107	1,810,422	5.806	0.054
4	15.1	2-Hydroxyethyl methacrylate (HEMA)	0.0107	117,022,400	375.291	3.5
5	15.9	Oxygen contained compound	0.0107	553,519	1.775	0.017
6	16.3	Methacrylate	0.0107	853,851	2.738	0.026
7	20.9	Ethylene glycol dimethacrylate (EGDMA)	0.0107	53,523,604	171.650	1.6
Total			0.0107	233,688,689	749.440	7.0

*1　Concentrations of VOCs were estimated based on Toluene response factor. Compounds out of calibration curve is calculated by extrapolation.

Table 4　　Region C：Analysis results of TD-GC/MS

Peak#	R.T. (min)	Compound	Sample mass (g)	Peak area	Detection amount (μg)	Amount generated from the sample (mass%)*1
1	6.6	Ethylene glycol	0.0107	4,195,187	13.454	0.13
2	8.8	Toluene	0.0107	239,521	0.768	0.0072
3	9.2	Ethyl methacrylate	0.0107	699,656	2.244	0.021
4	10.0	Methacrylic acid	0.0107	37,674,997	120.824	1.1
5	15.1	2-Hydroxyethyl methacrylate (HEMA)	0.0107	148,587,503	476.520	4.5
6	20.9	Ethylene glycol dimethacrylate (EGDMA)	0.0107	81,376,950	260.976	2.4
Total			0.0107	352,610,130	1130.821	11

*1　Concentrations of VOCs were estimated based on Toluene response factor. Compounds out of calibration curve is calculated by extrapolation.

Figure S15. Tabulated summary of TD-GC/MS results for outgassing from regions A, B, and C of S1 3DMIP, related to the thermal degradation of 3DMIP paragraph of the Results and Discussion section. HEMA and EGDMA were the dominant components across all regions; no CBD was detected. The findings validate the TG-MS results.

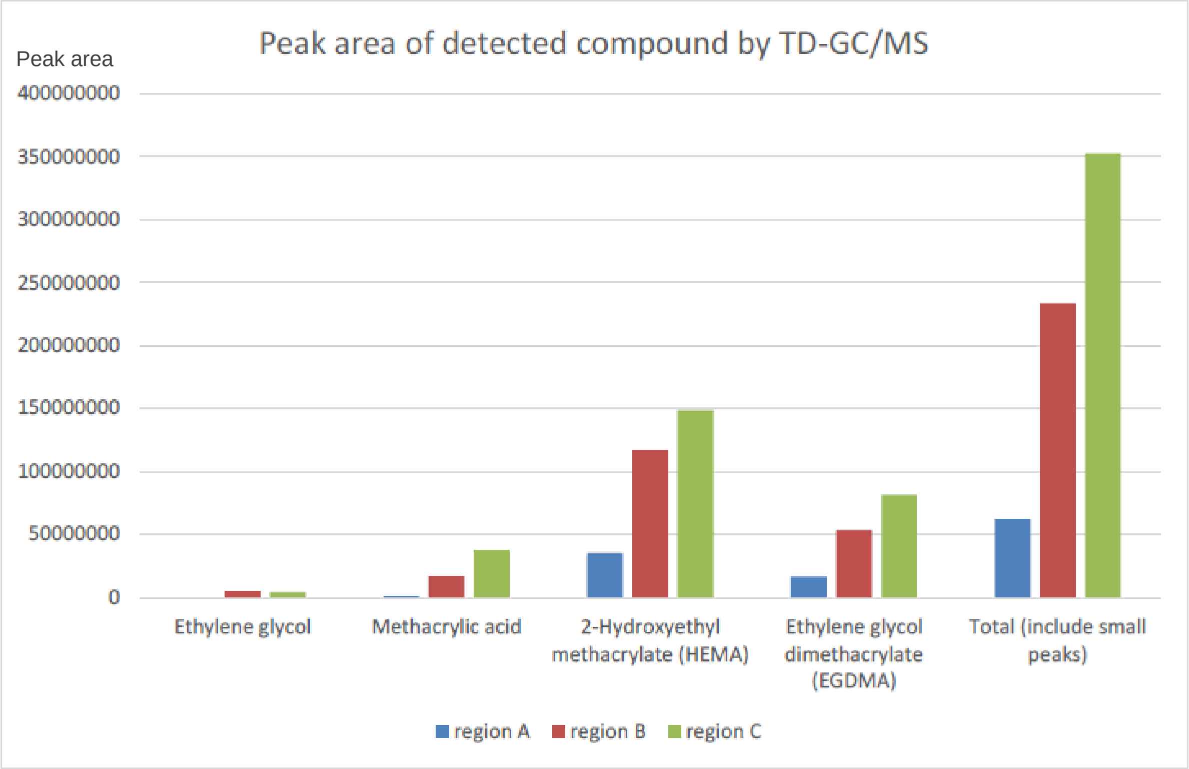

Figure S16. Bar diagram summarizing TD-GC/MS results for outgassing from regions A, B, and C of S1 3DMIP, illustrating the distribution of components across each temperature interval, related to the thermal degradation of 3DMIP paragraph of the Results and Discussion section.
